# High-resolution genome assembly and population genetic study of the endangered maple *Acer pentaphyllum* (Sapindaceae): implications for conservation strategies

**DOI:** 10.1093/hr/uhae357

**Published:** 2024-12-17

**Authors:** Xiong Li, Li-Sha Jiang, Heng-Ning Deng, Qi Yu, Wen-Bin Ju, Xiao-Juan Chen, Yu Feng, Bo Xu

**Affiliations:** CAS Key Laboratory of Mountain Ecological Restoration and Bioresource Utilization & Ecological Restoration and Biodiversity Conservation Key Laboratory of Sichuan Province, Chengdu Institute of Biology, Chinese Academy of Sciences, Chengdu, China; College of Life Sciences, University of Chinese Academy of Sciences, Beijing, China; CAS Key Laboratory of Mountain Ecological Restoration and Bioresource Utilization & Ecological Restoration and Biodiversity Conservation Key Laboratory of Sichuan Province, Chengdu Institute of Biology, Chinese Academy of Sciences, Chengdu, China; CAS Key Laboratory of Mountain Ecological Restoration and Bioresource Utilization & Ecological Restoration and Biodiversity Conservation Key Laboratory of Sichuan Province, Chengdu Institute of Biology, Chinese Academy of Sciences, Chengdu, China; College of Life Sciences, University of Chinese Academy of Sciences, Beijing, China; CAS Key Laboratory of Mountain Ecological Restoration and Bioresource Utilization & Ecological Restoration and Biodiversity Conservation Key Laboratory of Sichuan Province, Chengdu Institute of Biology, Chinese Academy of Sciences, Chengdu, China; CAS Key Laboratory of Mountain Ecological Restoration and Bioresource Utilization & Ecological Restoration and Biodiversity Conservation Key Laboratory of Sichuan Province, Chengdu Institute of Biology, Chinese Academy of Sciences, Chengdu, China; Wild Plants Sharing and Service Platform of Sichuan Province, Chengdu, China; CAS Key Laboratory of Mountain Ecological Restoration and Bioresource Utilization & Ecological Restoration and Biodiversity Conservation Key Laboratory of Sichuan Province, Chengdu Institute of Biology, Chinese Academy of Sciences, Chengdu, China; CAS Key Laboratory of Mountain Ecological Restoration and Bioresource Utilization & Ecological Restoration and Biodiversity Conservation Key Laboratory of Sichuan Province, Chengdu Institute of Biology, Chinese Academy of Sciences, Chengdu, China; CAS Key Laboratory of Mountain Ecological Restoration and Bioresource Utilization & Ecological Restoration and Biodiversity Conservation Key Laboratory of Sichuan Province, Chengdu Institute of Biology, Chinese Academy of Sciences, Chengdu, China; Wild Plants Sharing and Service Platform of Sichuan Province, Chengdu, China

## Abstract

*Acer pentaphyllum* Diels (Sapindaceae), a highly threatened maple endemic to the dry-hot valleys of the Yalong River in western Sichuan, China, represents a valuable resource for horticulture and conservation. This study presents the first chromosomal-scale genome assembly of *A. pentaphyllum* (~626 Mb, 2*n* = 26), constructed using PacBio HiFi and Hi-C sequencing technologies. Comparative genomic analyses revealed significant recent genomic changes through rapid amplification of transposable elements, particularly long terminal repeat retrotransposons, coinciding with the dramatic climate change during recent uplift of the Hengduan Mountains. Genes involved in photosynthesis, plant hormone signal transduction, and plant–pathogen interaction showed expansion and/or positive selection, potentially reflecting adaptation to the species’ unique dry-hot valley habitat. Population genomic analysis of 227 individuals from 28 populations revealed low genetic diversity (1.04 ± 0.97 × 10^−3^) compared to other woody species. Phylogeographic patterns suggest an unexpected upstream colonization along the Yalong River, while Quaternary climate fluctuations drove its continuous lineage diversification and population contraction. Assessment of genetic diversity, inbreeding, and genetic load across populations revealed concerning levels of inbreeding and accumulation of deleterious mutations in small, isolated populations, particularly those at range edges (TKX, CDG, TES). Based on these results, we propose conservation strategies, including the identification of management units and recommendations for genetic rescue. These findings not only facilitate the conservation of *A. pentaphyllum* but also serve as a valuable resource for future horticultural development and as a model for similar studies on other endangered plant species adapted to extreme environments.

## Introduction

The genus *Acer* L., commonly known as maples, is placed in the soapberry family, along with lychee and horse chestnut [1]. Comprising approximately 200 species, *Acer* is predominantly distributed across the temperate regions of the Northern Hemisphere, with a particular concentration in Asia. China stands as the modern center of *Acer* diversity, hosting over 100 species [2]. Maples are renowned for their distinctive palmate leaves and winged fruits (samaras), and hold significant ecological, economic, and cultural importance. Their applications span timber production, ethnomedicine, ecosystem services, and ornamental horticulture. Traditional medicinal uses include the consumption of young buds and leaves in teas for circulatory benefits, and the use of fruits for their purported anti-inflammatory and oral health properties [3, 4].

**Figure 1:**
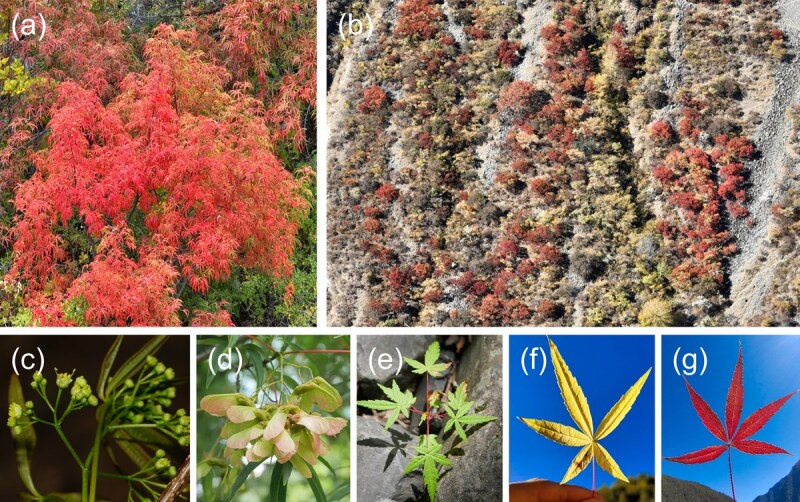
Photographs of *A. pentaphyllum.* (a) Adult tree. (b) Massive distribution along the Yalong River valley. (c) Flower. (d) Samara. (e) Seedling. (f) (g) Leaves in different colors during autumn

*Acer pentaphyllum* Diels is a unique species within the genus and the sole member of the series *Pentaphylla*. Endemic to the dry-hot valley of the Yalong River in western Sichuan Province, China, it grows at elevations ranging from 2100 to 3100 meters. This deciduous tree, reaching heights of up to 10 meters, is renowned for its striking autumnal transformation, with leaves shifting from green to yellow and finally to a vivid golden-red, creating breathtaking landscapes along the Yalong River valley during the late autumn and early winter months (Fig. 1). Such aesthetic qualities make it highly desirable for horticultural applications, particularly in ornamental landscaping, earning it acclaim as one of the most ornamentally appealing maples [5, 6]. This appreciation extends into Western gardens, where nurseries like Mendocino Maples praise its elegant form, distinctive narrow leaves, rare five-lobed pattern, and vibrant seasonal colors, making it an excellent choice for both landscape and container gardens (https://mendocinomaples.com/, last accessed 6 November 2024).

Cultivation efforts of *A. pentaphyllum* have been conducted by Western gardens since its first discovery in 1929 by Joseph Rock [7]. However, these efforts were obscured by its extreme rarity and presumed extinction due to a half-century absence from botanical records before its rediscovery in 1982 [7]. The species faces severe conservation challenges due to its narrow distribution range and habitat fragmentation caused by human activities such as road construction and grazing. Recent comprehensive surveys have confirmed its presence in only four counties: Yajiang, Kangding, Jiulong, and Muli, underscoring its limited distribution. Our field investigations also revealed alarmingly small population sizes in some locations. Consequently, *A. pentaphyllum* is classified as critically endangered (CR) in both the China Biodiversity Red List and the IUCN Red List of Threatened Species. Furthermore, the Sichuan government has designated it as a ‘plant species with extremely small population’ [8].

Beyond its conservation status, *A. pentaphyllum* holds significant scientific importance, particularly for studying the evolution of *Acer* genus and adaptation to the drought habitat in dry-hot valleys. However, despite numerous recent studies on this species [9–12], its evolutionary history, adaptation strategy, as well as genetic mechanisms underlying its endangered status remain poorly understood. This knowledge gap is primarily due to a lack of comprehensive genomic information, which has limited conservation efforts to traditional methods such as translocation. There is thus an urgent need for comprehensive research to inform effective conservation measures for this unique and valuable species.

Recent advancements in sequencing technologies have facilitated whole-genome studies across numerous *Acer* species, including *Acer yangbiense* [13], *Acer truncatum* [14], *Acer catalpifolium* [15], *Acer saccharum, Acer negundo* [16], *Acer pseudosieboldianum* [17], *Acer rubrum* [18] and *Acer palmatum* [19]. These genomic resources have provided unprecedented insights into the evolution and diversification of the *Acer* genus, shedding light on their adaptive strategies and phylogenetic relationships. Concurrently, conservation genomic studies combining whole-genome sequencing and population-level resequencing have significantly enhanced our understanding of the genetic mechanisms underlying population decline in many endangered plant species. Notable examples include studies on *Ostrya* [20], *Dipteronia* [21], and *Davidia* [22]. These investigations have revealed critical information about genetic diversity, inbreeding depression, and adaptive potential, which are essential for developing effective conservation strategies.

Here, we present the first high-quality, chromosome-level genome for *A. pentaphyllum* using the PacBio Single Molecule Real Time (SMRT) and next-generation sequencing technologies. To comprehensively elucidate the species’ genetic landscape, we employed an integrative approach combining comparative genomics with population genetics analyses. Our dataset encompasses 227 resequenced individuals collected across 28 remaining wild populations, representing an unprecedented sampling effort for this CR maple species. Utilizing this extensive genomic data, we conducted a series of in-depth analyses to address several key questions pertaining to the evolutionary history, adaptation, and conservation of *A. pentaphyllum*: (i) How has adaptation to the dry-hot valley environment been reflected in the genome? (ii) How have past climate changes influenced the colonization and demographic history of *A. pentaphyllum*? (iii) How can genomic and population genetic findings provide insights into the conservation of this CR species? By addressing these questions, this study not only advances our understanding of *A. pentaphyllum*’s biology and evolution but also provides crucial insights into developing evidence-based conservation strategies. Our findings will contribute to the scientific basis for preserving *A. pentaphyllum* and maintaining the rich biodiversity of its unique ecosystem.

**Figure 2:**
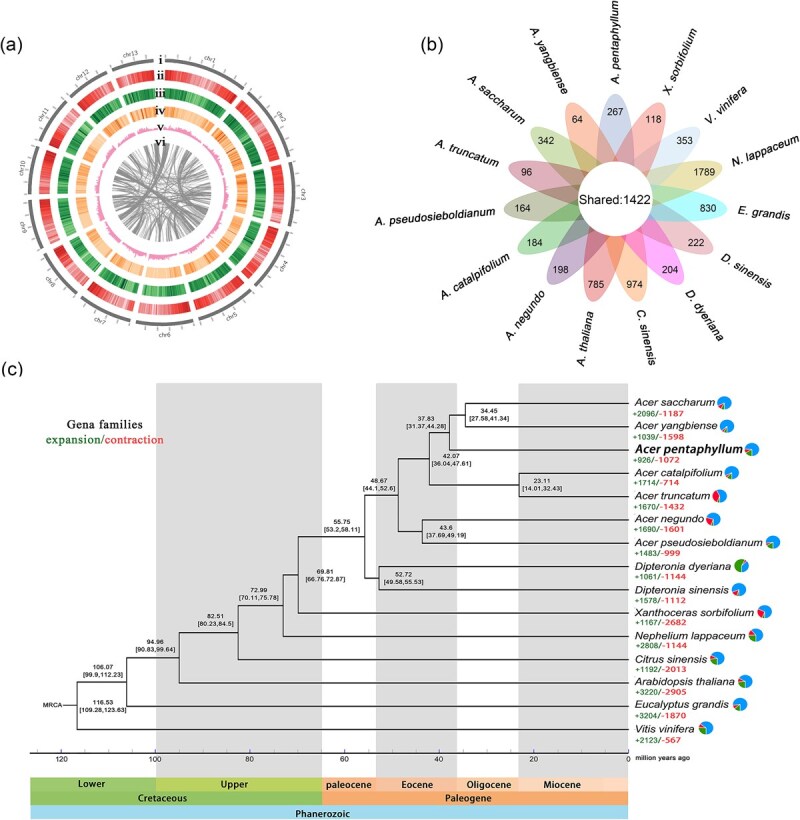
Genomic characteristics and genome evolution of *A. pentaphyllum*. (a) Circos plot of the genomic features. The circles, from outermost to innermost, show (i) thirteen pseudochromosomes, (ii) gene density, (iii) repeat element density, (iv) single nucleotide polymorphism (SNP) density for population genomic analysis, (v) GC content, and (vi) syntenic blocks. (b) Petal map of unique gene families and shared single-copy genes in 15 woody species. (c) A maximum likelihood tree of 15 Rosids species constructed based on 1422 single-copy orthologous genes. Gene families that expanded and contracted are displayed as numbers below each species of the tree, and values at the nodes represent the divergence times and 95% highest posterior density (HPD) intervals

## Results

### Genome sequencing, assembly, and assessment

We obtained a total of approximately 126.96 Gb (188.45×) sequencing data for the *de novo* assembly of *A. pentaphyllum*, including 30.12 Gb (44.47×) PacBio HiFi reads, 35.98 Gb (53.13×) Illumina short reads, and 60.86 Gb (89.85×) Hi-C sequencing data (Table S1). GenomeScope v1 [23] results suggested low heterozygosity rate (0.156%) and high repeat contents (69.59%) in the *A. pentaphyllum* genome. Utilizing the optimal assembly method, the draft assembly of *A. pentaphyllum* was ~662.38 million base pairs (Mb), which was similar to genome size estimated by the flow cytometry (630 Mb) and that calculated using 17-mers (677 Mb) (Figs S1–S2, Table S2, Appendix S1). Consistent with the results of the chromosome squashing (Fig. S3), in total 65 contigs (626.26 Mb, accounting for 94.55% of the total assembly) were anchored to the 13 pseudo-chromosomes of *A. pentaphyllum,* with a contig N50 of 25.06 Mb and a scaffold N50 of 48.21 Mb (Fig. 2a, Fig. S4, Table S3). Assessment of the completeness and consistency suggested that 99.99% of HiFi sequences could be mapped to the final assembly and 98.7% of complete benchmarking universal single-copy orthologs (BUSCO) groups (2294) were detected (Table S4), suggesting a high-quality reference genome for *A. pentaphyllum*.

### Genome annotation

We identified and annotated a total of 1 283 444 repetitive sequences, comprising 70.64% (~467.93 Mb) of the *A. pentaphyllum* genome. Among these, the most abundant elements were long terminal repeat retrotransposons (LTR-RTs), which made up 45.93% (~304.22 Mb) of the genomic content. The primary components were the *Copia* and *Gypsy* superfamilies, accounting for 20.80% (~137.77 Mb) and 13.89% (~92 Mb) of the assembled genome, respectively (Table S5). The distribution of LTR insertion times showed that most LTR-RTs have gradually accumulated in the *A. pentaphyllum* genome over the past 10 million years, with a peak of insertion activity occurring around 1.3 million years ago (Mya, Fig. S5). A total of 38 540 protein-coding genes were successfully annotated in the genome of *A. pentaphyllum* based on structural predictions and transcriptome data, spanning 146.18 Mb and accounting for 23.34% of the total genome. The mean lengths of gene region, transcript, and protein-coding region (CDS) were 3792, 1689, and 1425 bp, respectively (Table S6). Among the 38 540 genes predicted in the *A. pentaphyllum* genome, functions of 36 336 genes (accounted for approximately 94.28%) were annotated using four databases (Fig. S6, Table S7). Moreover, we identified 10 972 non-coding RNAs (ncRNAs), accounting for 0.44% (~2914.25 kb) of the *A. pentaphyllum* genome (Table S8).

### Genomic phylogenetic evolution and signatures of positive selection

Overall, we identified 460 262 homologous genes belonging to 30 421 gene families from 15 Rosids genomes, among which 1422 were single-copy orthologous genes shared by all species, and 267 gene families containing 1572 genes were species-specific to *A. pentaphyllum* (Fig. 2b, Fig. S7, Table S9). The dated phylogenetic tree showed that *A. pentaphyllum* is sister to a clade consisting of *A. saccharum* and *A. yangbiense*, with the divergence time estimated at approximately 37.83 Mya (95% highest posterior density, HPD: 44.28–31.37 Mya; Fig. 2c). Furthermore, *Acer* and *Dipteronia* diverged from each other in the late Paleocene–early Eocene boundary (~55.75 Mya, 95% HPD: 53.2–58.11, Fig. 2c).

**Figure 3:**
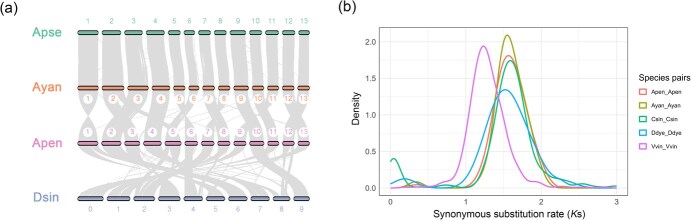
(a) Syntenic blocks among *Acer* and *Dipteronia* species. (b) Frequency distribution of *K*_s_ for paralogous genes within *A. pentaphyllum* (Apen), *A. yangbiense* (Ayan), *Citrus sinensis* (Csin), *Dipteronia dyeriana* (Ddye), and *Vitis vinifera* (Vvin)

A total of 926 expanded and 1072 contracted gene families in *A. pentaphyllum* were identified and annotated (Fig. 2c). Gene Ontology (GO) enrichment analysis of the expanded genes revealed significant enrichment (*P*-adjust, *Q* < 0.05) in macromolecular metabolism (GO:0019318, GO:0044238), cytochrome synthesis (GO:0016168), photosynthesis (GO:0009767, GO:0009772), ATP synthesis (GO:0015986), heme transport (GO:0015886, GO0015232), and ion transport (GO:1902600), among others (Fig. S8a, Table S10). Kyoto Encyclopedia of Genes and Genomes (KEGG) analysis identified 13 significantly enriched pathways (*Q* < 0.05), primarily associated with photosynthesis (ko00195), plant hormone signal transduction (ko04075), glyoxylic acid and dicarboxylic acid metabolism (ko00630), plant and pathogen interaction (ko04626), monoterpenoid biosynthesis (ko00902), and other metabolic pathways (Fig. S8b, Table S11). In addition, we identified 108 putative positively selected genes (*Q* < 0.05) in the *A. pentaphyllum* genome through calculating the ratio of non-synonymous substitutions to synonymous substitutions (*K*_a_/*K*_s_). These positively selected genes are significantly enriched (*Q* < 0.15) in biological processes related to abiotic stress, response to plant hormones, and catalytic activity (Fig. S8, Table S12).

### Analysis of collinearity and whole-genome duplication

Intragenomic collinearity analysis detected a total of 3381 colinear gene pairs on 290 high-quality colinear blocks (gene pairs ≥4) within the *A. pentaphyllum* genome using JCVI, accounting for 8.77% of the total genes. When compared to the genomes of the two *Acer* species and one *Dipteronia* species, *A. pentaphyllum* contained fewer proportion of intragenomic collinear gene pairs (*A. yangbiense*: 9.65% vs, *A. pseudosieboldianum*: 11.93%, and *Dipteronia sinensis*: 10.46%). Aside from a few cases of chromosome fusion or fission (such as chromosomes 2 and 6 in *A. yangbiense* and chromosome 5 in *A. pentaphyllum*), the distribution and arrangement of long collinear blocks among the three *Acer* species are relatively consistent across 13 chromosomes. Moreover, these blocks revealed a nearly 1:1 collinearity depth ratio (Fig. 3a, Fig. S10), indicating that there was no significant copy number variation in the chromosomes of *A. pentaphyllum* since diverging with other *Acer* species. In contrast, the arrangement of collinear blocks between *A. pentaphyllum* and *D. sinensis* is more disordered (Fig. 3a, Fig. S10), which aligns with their more distant positions in the phylogenetic tree (Fig. 2c). Additionally, *K*_s_ distribution of colinear genes within species showed only one major peak in *A. pentaphyllum*, consistent with *Vitis vinifera*, *Citrus sinensis,* and other Sapindaceae species (Fig. 3b). This pattern suggests similar evolutionary histories among these species, with no evidence of recent whole-genome duplications (WGDs) other than the hexaploidization shared by core eudicots [24].

### Field studies, population structure, and phylogenetic relationships

Through field investigations, we found a total of ~1.65 × 10^4^  *Acer pentaphyllum* individuals within the entire distribution areas, including ~7200 adult trees (Table S13). The TKX, MDG, TZC, ZQG, CDG, and MLC populations show extremely small numbers of individuals (<100) and mature trees (<50), whereas the AJ and ZD populations in Muli County are relatively large, with over 2000 individuals and more than 1000 mature trees (Table S13). *Acer pentaphyllum* typically grows on mid-slopes of valleys, occurring between sclerophyllous evergreen broadleaved forests and deciduous broad-leaved forests. Despite the large population size, we found severe habitat fragmentation and discontinuous distribution among neighboring populations, which were separated by either extremely cold climates in high mountains, extremely arid habitats, or farmlands in valley bottoms.

A total of ~3.01 Tb resequencing base pairs were generated for 227 *A. pentaphyllum* accessions from 28 wild populations, with an average sequencing depth of approximately 26× and an average heterozygosity of 0.16% (Table S14). After SNP calling and filtering, 2 757 997 high-quality SNPs were retained (Dataset 3). An annotation of SNPs showed that ~61.57% of SNPs were located in the intergenic regions while the remaining ~38.43% were found in genic regions, which encompass exonic (7.27%), splicing (0.04%), stop-gain (0.23%), nonsynonymous (4.42%), UTR5/3 (0.84%), intronic (10.83%), and upstream/downstream (21.02%) regions (Table S15).

**Figure 4:**
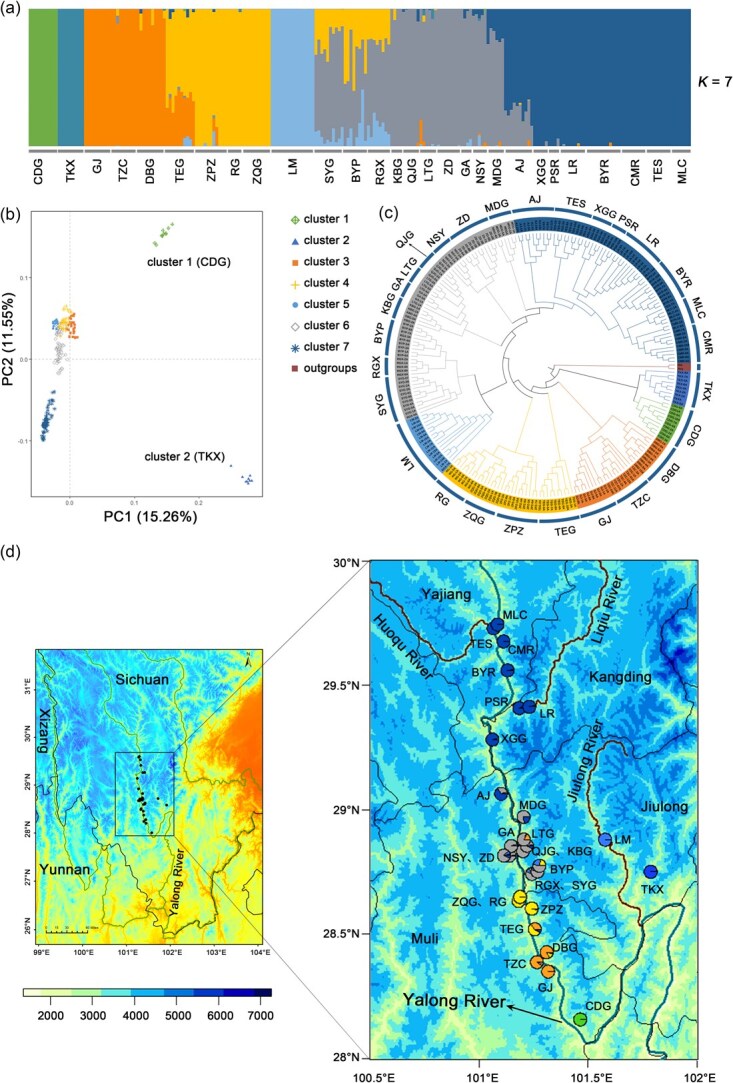
Genetic structure and phylogenetic relationships of the 28 populations of *A. pentaphyllum.* (a) Population genetic structure at *K* = 7. (b) PCA plot of *A. pentaphyllum* based on neutral loci. (c) The phylogenetic tree of 227 *A. pentaphyllum* individuals was constructed based on SNP Dataset 4. (d) Geographic distribution maps of ADMIXTURE clusters for the 28 populations of *A. pentaphyllum* at *K* = 7. The colors of the populations in (b), (c), and (d) correspond to the genetic clusters in (a)

The admixture analysis based on unlinked and intergenic sites (Dataset 4) revealed distinct intraspecies genetic structures among populations of *A. pentaphyllum*, with seven clusters (*K* = 7) at the lowest cross-validation (CV) error (Fig. 4a, Table S16). Individuals from cluster 1 (CDG), cluster 2 (TKX), cluster 5 (LM), and cluster 7 showed relatively purer ancestry compared to other *A. pentaphyllum* clusters, especially those from cluster 6. This group is distributed at the center of the overall distribution across the river and showed higher levels of mixed genetic ancestry (Fig. 4a and d). Additional subclusters were detected at higher *K* values (Fig. S11). Interestingly, individuals from TES clustered with those from Yajiang and Kangding counties when *K* < 8, but as *K* increases, TES shared a similar genetic ancestry to AJ (Fig. S11), despite the considerable geographic distance between these two populations (~74 km). PCA analysis revealed clear separations of the CDG and TKX populations from all others based on PC1 and PC2, which accounted for 15.26% and 11.55% of the total variance, respectively (Fig. 4b).

The results of the phylogenetic analysis were consistent with admixture and PCA analysis, revealing that individuals from most sample locations clustered into distinct monophyletic clades, except a few from clusters 6 and 7 (e.g. RGX, MLC; Figs 4c and S11). Notably, the phylogenetic positions of the 28 populations on the ML tree were geographically located almost completely opposite to the flow direction of the Yalong River (Figs 4c, 4d, and S12). Specifically, individuals from CDG and TKX, situated in the lower reaches of *A. pentaphyllum*’s entire distribution range*,* diverged first at the base of the tree. This was followed by the divergence of individuals from Muli and Jiulong counties, while individuals from Kangding and Yajiang counties diverged most recently (Fig. 4c and d). However, the TES population from Yajiang County, located geographically closest to MLC at the northern edge of the overall distribution, diverged earlier compared to the other populations from Yajiang and Kangding counties, except the AJ population, which is the basal group of cluster 7 (Figs 4c, 4d, and S13). Additionally, several populations located on opposite sides of the river, such as DBG-TZC-GJ, ZPZ-RG-ZQG, and GA-LTG-QJG, were also clustered together on the tree (Fig. 4d).

### Genome-wide diversity and inbreeding

Genome-wide linkage disequilibrium (LD) decay analysis revealed remarkable variation among the *A. pentaphyllum* populations. The decay of LD reached half of its maximum average *r*^2^ at distances ranging from ~16.78 kb in the AJ population to ~191.84 kb in the GJ population (Fig. S13). This substantial variation in LD decay rates reflects the diverse demographic histories of these populations. The genetic diversity (*π*) of this narrow endemic species was found to be relatively low (1.212 ± 1.058 × 10^−3^) compared to other threatened woody plant species with available genetic diversity information (Fig. S14, Table S17). It was lower than *A. yangbiense* (3.13 × 10^−3^) and *Rhododendron griersonianum* (1.94 × 10^−3^; [25]), but slightly higher than *Cercidiphyllum japonicum* (1.1 × 10^−3^; [26]), *Manglietiastrum sinicumand* (1.16 × 10^−3^; [27]), and *Populus ilicifolia* (8.3 × 10^−4^; Fig. S14, Table S17; [28]). Among the populations within *A. pentaphyllum,* the RG population had the highest genetic diversity (1.083 ± 1.071 × 10^−3^), while the TKX population had the lowest genetic diversity (5.74 ± 7.65 × 10^−4^, Table 1). The overall *F*_ST_ analysis results indicated a positive correlation between geographical distance and genetic distance among population pairs of *A. pentaphyllum* (Pearson’s *R* = 0.4495, Fig. S15). Consistent with the closer evolutionary relationships (Fig. 4c) exhibited between nearby populations of *A. pentaphyllum*, neighboring populations, even those located on opposite sides of the Yalong River, often showed lower *F*_ST_ (Fig. S16, Tables S18–S19), suggesting low genetic differentiation between them. Moreover, consistent with the results of population genetic structure and PCA (Fig. 4), TKX (mean pairwise *F*_ST_ = 0.48) and CDG (mean pairwise *F*_ST_ = 0.28) populations showed a significant differentiation from the other populations of *A. pentaphyllum* (Fig. S16, Table S18).

**Table 1:** The genetic diversity (*π*), Tajima’s *D*, and inbreeding coefficients (*F*_IS_) for each cluster and population of *A. pentaphyllum*.

**Cluster**	**Population**	**County**	***π***	**Tajima’s *D***	***F***_**IS**_
1 *π* = 0.813 ± 0.932 × 10^−3^ Tajima’s *D* = 0.315873	CDG	Muli	0.000813	0.316215	0.29268
2 *π* = 0.574 ± 0.765 × 10^−3^ Tajima’s *D* = 0.30531	TKX	Jiulong	0.000574	0.305749	0.4051
3 *π* = 1.054 ± 0.959 × 10^−3^ Tajima’s *D* = 0.359512	GJ	Muli	0.000851	0.264326	0.2892
	TZC	Muli	0.000863	0.06625	0.32
	DBG	Muli	0.001022	0.275735	0.294
4 *π* = 1.094 ± 1.018 × 10^−3^ Tajima’s *D* = 0.434142	TEG	Muli	0.001036	0.323331	0.136
	ZPZ	Muli	0.000932	0.389089	0.2035
	RG	Muli	0.000832	0.317693	0.2195
	ZQG	Muli	0.000878	0.355773	0.2276
5 *π* = 0.873 ± 0.941 × 10^−3^ Tajima’s *D* = 0.612627	LM	Jiulong	0.000873	0.612742	0.1702
6 *π* = 1.172 ± 1.040 × 10^−3^ Tajima’s *D* = 0.257316	SYG	Jiulong	0.001029	0.284683	0.1954
	BYP	Jiulong	0.000994	0.277248	0.2615
	RGX	Jiulong	0.001083	0.491352	0.1009
	LTG	Jiulong	0.001053	0.200623	0.2128
	QJG	Jiulong	0.001028	0.115578	0.0941
	KBG	Jiulong	0.000993	0.084268	0.0891
	ZD	Muli	0.001046	0.275483	−0.0242
	GA	Muli	0.001024	0.215596	0.0627
	NSY	Muli	0.00108	0.062116	0.0068
	MDG	Jiulong	0.000969	−0.04696	0.1447
7 *π* = 1.001 ± 0.958 × 10^−3^ Tajima’s *D* = 0.354932	AJ	Muli	0.001024	0.280309	0.1815
	XGG	Kangding	0.000973	0.217838	0.2285
	PSR	Kangding	0.000701	0.32515	0.1382
	LR	Kangding	0.000895	0.224851	0.2423
	BYR	Yajiang	0.000953	0.229346	0.2774
	CMR	Yajiang	0.000875	0.267159	0.2219
	TES	Yajiang	0.000737	0.2388	0.2747
	MLC	Yajiang	0.000917	0.151434	0.1701

The estimation of kinship coefficient among individuals identified a total of 67 pairs of individuals as less than third-degree relatives from the 7 clusters of *A. pentaphyllum.* This included 11 pairs categorized as duplicate or monozygotic twins (Dup/MZ Twins), 22 pairs as second-degree, 33 pairs as parent–offspring (PO), and 1 pair of full siblings (FS). Notably, the small-size populations, including the TKX, CDG, TZC, LM, and TES, had a higher proportion (64.18%) of closely related individual pairs, most of which were estimated to be second-degree or PO relatives (Table S20). Similar, higher inbreeding coefficients (*F*_IS_) were observed in the TKX (0.4051), TZC (0.32), and TES (0.29), while most populations of lineage 6 exhibited lower *F*_IS_ (0.096, Table 1).

PLINK identified a total of 192 587 runs of homozygosity (ROHs) across all 227 individuals of *A. pentaphyllum,* averaging approximately 848 ROHs per individual. The mean ROH length (*L*_ROH_) was 168.39 kb, with the longest segment measuring 1.583 Mb (7240 SNPs) found on XGG-1 (Table S21). The fraction of ROH in the genome (*F*_ROH_) was significantly higher in the BYR (0.35 ± 0.15), TZC (0.32 ± 0.05), GJ (0.30 ± 0.06), and TKX (0.29 ± 0.07) populations, each characterized by a high number of short ROHs (*L*_ROH_ < 200 kb), with 7000, 5371, 6552, and 8348 segments, respectively (Table S22). Short ROHs are typically indicative of ancient inbreeding, where recombination over many generations has broken down longer stretches of homozygosity into smaller segments [29]. These short segments accounted for approximately 76.12% of all detected ROHs and contributed the highest proportion (60.52%) of the cumulative ROH length, which significantly exceeded the contributions of large (*L*_ROH_ > 400 kb, 6.48%) and medium segments (200 kb < *L*_ROH_ ≤ 400 kb, 33%, Table S22), implying high levels of ancient inbreeding in *A. pentaphyllum*. However, consistent with the *F*_IS_ estimation, the *F*_ROH_ of individuals from cluster 6 was very low (mean *F*_ROH_ < 0.15), indicating reduced inbreeding within this cluster, which is located in the central region of the *A. pentaphyllum* distribution area (Table 1, Table S21, Fig. 6a).

### Demographic history inference and species distribution modeling

**Figure 5:**
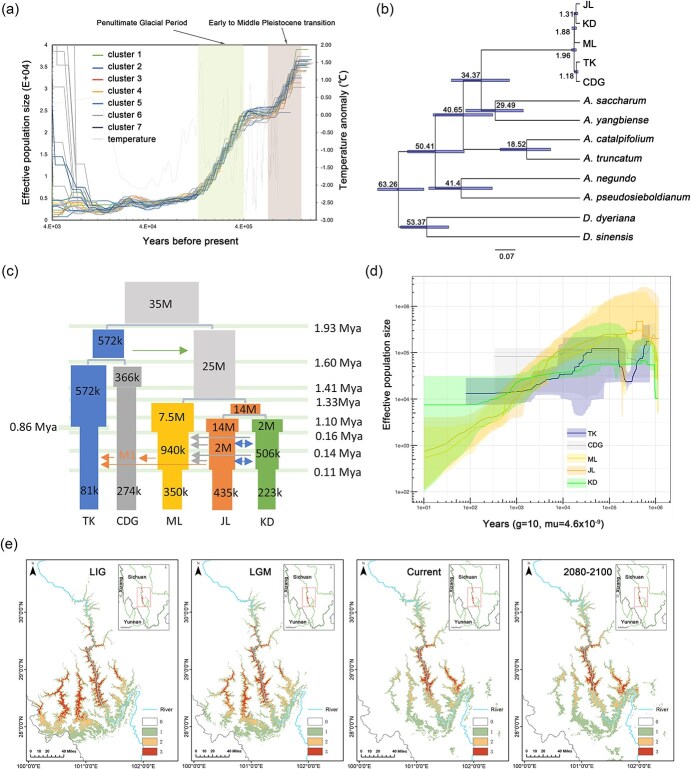
Demographic history of *A. pentaphyllum* inferred by different methods. (a) Historical changes in effective population size (*N*_e_) through time plots for *A. pentaphyllum* based on PSMC modeling, with a generation time of 10 years and a mutation rate ($\mu$) of 4.7e-9. The Early to Middle Pleistocene transition: ~1.6–0.7 Mya; the Penultimate Glacial Period: ~0.36–0.13 Mya. (b) Dated phylogeny of clusters within *A. pentaphyllum* and six *Acer* species, with two *Dipteronia* species as outgroups. (c) Illustration of the best-fit model with point estimations of the parameters. (d) Estimation of changes in effective population size changes over time for each cluster using Stairway Plot 2. Colored shadings represent 95% confidence intervals calculated from 200 bootstrap replicates. (e) Species distribution modeling for four time periods: LIG, LGM, Current, and Future (2080–2100 under SSP126). Habitat suitability is divided into four categories, numbers 0 to 3 represent low (0–0.18), moderate (0.18–0.39), high (0.39–0.74), and very high suitability (0.74–0.99) zones. The blue line denotes the Yalong River

The demographic history inferred by PSMC suggested that *A. pentaphyllum* experienced two severe population declines within the past million years. The earliest population decline began around 1.4 Mya and lasted until about 0.8 Mya, while the more recent decline started around 0.36 Mya and continued until approximately 0.1 Mya (Fig. 5a). Following the most recent bottleneck, *A. pentaphyllum* has maintained a stable effective population size (*N*_e_) until recently. The *N*_e_ of *A. pentaphyllum* was further estimated to be 5.78 × 10^4^ based on the number of SNPs and samples (Table S13).

**Figure 6:**
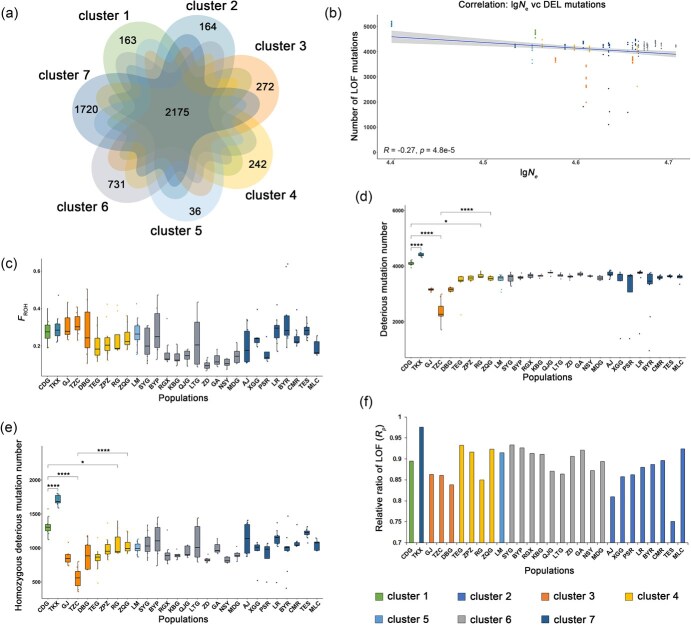
Assessment of inbreeding and genetic load in 28 populations of *A. pentaphyllum*. (a) Venn diagram of private or shared deleterious mutations among clusters of *A. pentaphyllum* at *K* = 7. (b) Correlation between the number of extreme deleterious mutations (LOF) and effective population size (*N*_e_) calculated by the formula *N_e_* = *θ/(4 μ)* across the 28 populations of *A. pentaphyllum*. (c) Boxplot showing *F*_ROH_ of individuals grouped by populations. (d) Number of deleterious mutations, including DEL, RADICAL, and LOF sites. (e) Number of homozygous deleterious mutations, which are calculated as 2 × homozygous deleterious genotypes. (f) The relative ratio (${R}_p$) of the mean derived alleles frequency ($p$) for LOF mutations

To explore the comprehensive demographic histories including lineage divergence, gene flow, and population size changes through time, we employed coalescent population simulation and composite likelihood method in fastsimcoal2 [30]. The populations were grouped into five major clusters according to their geographic distribution and results of phylogenetic analysis (CDG: cluster 1; TKX: cluster 2; ML: clusters 3–5; JL: cluster 6, KD, cluster 7). The priority distributions of divergence time were estimated from the 1422 single copy orthologous genes in the comparative genomic analysis. Results showed that the crown age of *A. pentaphyllum* was dated to the early/middle Pleistocene, ca. 1.96 (95% HPD: 2.82–1.18) Mya, with the clusters diverged from ca. 1.88–1.18 Mya (Fig. 5b).

Comparison of likelihood values among 10 demographic scenarios revealed that Model 10 exhibited the highest likelihood (Fig. 5c, Figs S17–S18, Table S23). According to this model, the most recent common ancestor (MRCA) of CDG and TKX diverged from the MRCA of the other three clusters approximately 1.93 Mya. Subsequently, CDG and TKX separated around 1.60 Mya, and thereafter experienced population contraction around 1.41 Mya and 0.86 Mya, respectively. The remaining three clusters diverged around 1.33 Mya and 1.10 Mya. Following divergence, ML, JL, and KD underwent two distinct population contractions at approximately 0.16 Mya and 0.11 Mya, respectively (Fig. 5c). The earliest gene flow occurred from the ancestor of TKX and CDG to the ancestor of the remaining lineages during the early Pleistocene (1.93–1.60 Mya). After the lineage divergence, gene flow was detected between JL and KD, and from KD to JL and ML, spanning from 0.16 Mya to 0.11 Mya. Additionally, gene flow from ML to TKX and CDG, as well as from JL to ML, and from CDG to TKX was observed between 0.14 Mya and 0.11 Mya (Figs 5c and S18). In addition, population size changes through time inferred based on SFS by StairwayPlot2 revealed similar patterns for these clusters (Fig. 5d, Table S24).

The species distribution modeling across four periods demonstrated a clear trend of contraction in the south since the Last Interglacial (LIG) and slight northward shift since the Last Glacial Maximum (LGM) in suitable habitat areas of *A. pentaphyllum* (Fig. 5e). In the current period, the predicted suitable habitats are restricted and align with the current distribution, with high-suitability zones primarily along the middle and upper reaches of the Yalong River. Future projections suggested that suitable areas will continue to decrease and become more localized in the upper reaches of the river, indicating a northward shift and range contraction likely driven by climate change (Fig. 5e).

### Evaluation of inbreeding and mutation load

After filtering out SNPs with reference alleles inconsistent with ancestral states in the *A. pentaphyllum* genome, 67 769 sites from protein-coding regions were retained for the evaluation of deleterious mutations. A total of 23 290 mutations were identified and classified into three groups, of which 19 699 (84.58%) were ‘deleterious’ (DEL) annotated by SIFT4G, 5600 (24.04%) were detected as ‘radical missense substitution’ (RADICAL) with Grantham score > 150, and 1499 (6.44%) were loss-of-function (LOF) mutations identified through SNPEFF (Table S25). The seven clusters shared 2175 deleterious mutant sites (9.34% of the total), with at least one individual from each cluster carrying deleterious derived alleles at these sites. Cluster 5 (LM, 16 individuals) exhibited the fewest unique deleterious mutations (36, 0.15%), while cluster 7 (64 individuals) had accumulated the most unique deleterious mutations (1720, 7.39%) (Fig. 6a). Moreover, among the 23 290 mutations, 10 364 (44.50%) were homozygous in at least one individual (Table S26). A considerable number of observed homozygous deleterious mutations (1712, 16.85% of the total homozygous sites) were shared by more than 50 individuals (Table S26).

We found a negative correlation between the number of deleterious mutations and effective population size among the 28 populations of *A. pentaphyllum* (DEL vs *N*_e_: Pearson’s *R* = −0.27, *P* < 4.8e–5; LOF vs *N*_e_: Pearson’s *R* = −0.2, *P* < 2.5e–3; Figs 6b and S19), suggesting that smaller populations tend to accumulate more deleterious mutations. A similar trend was observed with census population size, although it was less pronounced and not as significant (DEL vs *N*_c_: Pearson’s *R* = −0.077, *P* = 0.25; LOF vs *N*_c_: Pearson’s *R* = −0.15, *P* = 0.023; Fig. S20). For example, samples from CDG and TKX had higher inbreeding (*F*_ROH_: 27.64% and 29.87%, Fig. 6c, Table S21) than the average of all populations, and carried significantly more deleterious derived alleles (DEL: 3518 and 3800; RADICAL: 913 and 967; LOF: 236 and 277) compared to the remaining individuals of *A. pentaphyllum* (Fig. 6c, Fig. 6d, Fig. S21, and Table S27). TKX and CDG carried 31–70% more homozygous DEL alleles, 31–85% more homozygous RADICAL alleles, and 25–59% more homozygous LOF alleles than average, indicating high genetic load in these two populations of *A. pentaphyllum* (Fig. 6e, Fig. S22, Table S27). However, despite having a high *F*_ROH_ (31.84%), with small population size (only 30 mature individuals) and low nucleotide diversity (8.63 ± 8.88 × 10^−4^, Table 1, Table S13), the TZC population from cluster 3 exhibited the fewest number of deleterious mutations (DEL: 2045; RADICAL: = 561; LOF: 133) and most of them were present in a heterozygous state (DEL: 1092, 53.40%; RADICAL: 301, 53.65%; LOF: 71, 53.38%) (Fig. 6c, Fig. 6d, Fig. S23, Table S27). However, in cluster 6, some populations (e.g. LTG) showed high *F*_ROH_ and numerous deleterious mutations, while others (e.g. BYP) displayed high *F*_ROH_ but few deleterious mutations. Additionally, some populations (e.g. GA) had low *F*_ROH_ but many deleterious mutations (Fig. 6c, Fig. S21, Table S27). These varying patterns indicate that different demographic histories and isolation levels among these populations with different local habitats have resulted in contrasting levels of inbreeding and genetic load, despite their central distribution within the overall range.

The relative ratio (${R}_p$) of the mean derived alleles frequency reflects the strength of deleterious mutation purging for each population (Fig. S24a and Table S28). By calculating ${R}_p$ for three types of mutation, all populations of *A. pentaphyllum* exhibited similarly low efficiency (mean ${R}_p$ =1.01) in purging DEL deleterious mutations (Fig. S24a and Table S28). TKX showed the strongest purging ability against RADICAL (${R}_p$ = 0.91, Fig. S24b) mutations but the weakest ability to remove extreme deleterious mutations (LOF: ${R}_p$ = 0.98, Fig. 6f), while the TES population excelled in removing RADICAL (${R}_p$ = 0.93) and LOF (${R}_p$ = 0.75) sites (Fig. 6f, Fig. S24b, and Table S28).

**Figure 7:**
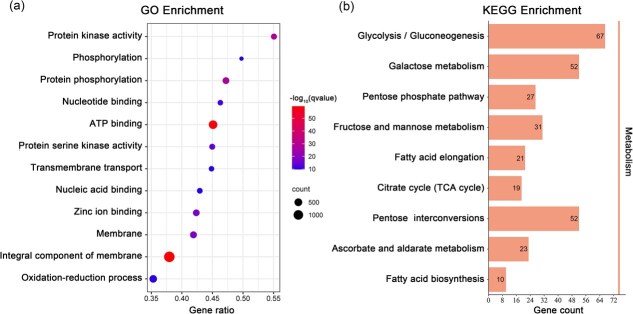
GO (a) and KEGG (b) enrichment diagram of genes containing deleterious mutations in *A. pentaphyllum*

GO enrichment analysis of genes containing deleterious mutations in *A. pentaphyllum* revealed significant enrichment (*Q* < 0.05) in the structure and function of membranes (GO: 0016021, GO: 0016020, GO: 0022857), ATP binding (GO: 0005524), protein activity (GO: 0004672, GO: 0004674, GO: 0016887, GO: 0016301, GO: 0004252), molecular binding (GO: 0008270, GO: 0000166, GO: 0003676, GO: 0046872), response to heat (GO:0009408), recognition of pollen (GO: 0048544), among others (Fig. 7a and Table S29). Further KEGG pathway analysis for these loci showed that they were mainly enriched (*Q* < 0.5) in carbohydrate and fatty acid metabolisms, such as the glycolysis/gluconeogenesis (ko00010), galactose metabolism (ko00052), fatty acid elongation (ko00062), and TCA cycle (ko00020) (Fig. 7b and Table S30).

## Discussions

### The evolving genome of the endangered maple tree

*Acer pentaphyllum* is one of the most elegant maples in the world, possessing significant horticultural potential due to its distinctive aesthetic traits. Beyond its ornamental value, the species holds great scientific importance due to its remarkable adaptability, particularly its drought tolerance, and its extreme rarity in nature. However, its small population size and endangered status underscore the urgent need for comprehensive research. Until recently, the lack of genomic data and a detailed understanding of its genetic diversity distribution have impeded progress in conservation efforts for this species. In this study, we presented for the first time a high-quality chromosomal-scale genome assembly for the narrow endemic *A. pentaphyllum* (~626 Mb) constructed using PacBio long HiFi reads and Hi-C high-accuracy sequencing. Taken together previously published *Acer* genomes, we found that abundant repetitive elements are a shared characteristic within the genus. For instance, repetitive sequences comprise 70.64% of the *A. pentaphyllum* genome (Table S5), similar to *A. yangbiense* (68.9% in a ~ 666 Mb genome; [13]) and *A. pseudosieboldianum* (76.84% in a ~ 690.24 Mb genome; [17]). Notably, all these species have undergone only the ancient γ hexaploidization event shared by core eudicots, with no evidence of recent WGDs.

However, the *A. pentaphyllum* genome exhibits evidence of significant recent genomic changes through rapid amplification of transposable elements (TEs), particularly long terminal repeat retrotransposons (LTR-RTs). This amplification is evident from the presence of short syntenic blocks in the genome over the past 10 million years (Fig. S5). Notably, this time frame coincides with a period of extensive *in situ* speciation in the Hengduan Mountains [31]. This burst of speciation was likely triggered by the rapid and recent uplift of the Hengduan Mountains and the formation of its distinctive landscape, characterized by deep valleys and steep slopes, occurring between the late Miocene and late Pliocene [31,32]. The dramatic changes in climate and environment associated with this geological activity may have been a driving force behind the burst of TE activity in the *A. pentaphyllum* genome. This recent surge in transposon proliferation has played a crucial role in shaping the current genomic landscape of *A. pentaphyllum* [33,34]. Such genomic restructuring may have significantly contributed to increased genomic variation and enhanced adaptability, underscoring the importance of TEs in the species’ evolutionary dynamics.

*Acer pentaphyllum* thrives in the dry-hot valley of the middle Yalong River, characterized by highly distinct wet and dry seasons. The winter months (November to April), experience minimal rainfall, accounting for less than 6% of the annual precipitation, with temperatures dropping to −15.6°C. In contrast, summer temperatures can soar to 31.7°C due to the valley’s intense heat concentration [10]. Comparative genomic analyses revealed that several genes involved in photosynthesis, plant hormone signal transduction, and plant–pathogen interaction were selectively expanded and/or positively selected in the genome during the processes of evolution (Fig. S9, Table S10, Table S11, and Table S12). These pathways play essential roles in plant development and adaptation. For example, most of the genes significantly enriched in the plant hormone signal transduction pathway were identified as encoding auxin-induced/responsive proteins, which have been reported to regulate various development and stress responses in plants [35,36]. Additionally, several expanded genes involved in the plant–pathogen interaction pathway can enhance defense responses against pathogens, boosting plant survival under biotic stress conditions [37]. Thus, these expanded and positively selected genes are potential candidates for adaptation of *A. pentaphyllum* to high-temperature and low-humidity valley environments.

### Quaternary climate fluctuation drove lineage diversification and population contraction of A*. pentaphyllum*

It has been widely recognized that the uplift of the southeastern margin of the Qinghai-Tibetan Plateau drove *in situ* diversification of many taxa [38]. The interplay among East Asia monsoon activities, regional uplift, glacial–interglacial cycles, and the evolution of drainage systems has shaped diverse evolutionary histories of species endemic to this region. For example, the population phylogeographic structure and genetic variation of *Buddleja crispa* perfectly reflect the putative Paleo Red River drainage pattern [39], while drainage isolation and Quaternary climate oscillations shape the genetic structures of *Tuber indicum* complex [40]. In our case, distributing in the deep valleys surrounded by high mountains, a subconscious colonization route of *A. pentaphyllum* is thought to be from the upstream to the downstream of the Yalong River, because wind may not be able to facilitate its seeds to fly over the high mountains but water is able to disperse the seeds for at least 250 km [41,42]. Interestingly, our population genomic analysis revealed a contrast pattern: CDG and TKX, representing the most downstream populations of *A. pentaphyllum* positioned the basal of the phylogenetic tree, while other upstream populations possessed derived lineages, exhibiting an upstream colonization before/during the lineage diversification of this species (Fig. 4c).

The Yalong River valley is a typical dry-hot valley shaped by the uplift of the southeastern margin of the Qinghai-Tibetan Plateau and subsequent activities of East Asia monsoon [43–45]. While an alpine climate prevails above 3000 meters and extremely arid and hot conditions dominate below 2000 meters, a mixed broadleaf forest thrives in between (2000–3000 meters), providing the primary habitat for *A. pentaphyllum*. Similar to other dry-hot valleys in southern Hengduan Mountains, where the establishment of the vegetation patterns could be traced back to Pliocene, the Yalong River valley exhibits warm affinity in the south and cool-temperate affinity in the north [46]. The divergence between *A. pentaphyllum* and its close relatives dated back to Mid-Miocene [47,48], while the lineage diversification of *A. pentaphyllum* was dated back to early Pleistocene (*c.* 2–1 Mya; Fig. 5c). The warm and humid climate before Mid-Pliocene may facilitate its northward colonization during the expansion of warm-temperate mixed forests [49,50]. However, the intensify of the East Asia monsoon during Late Pliocene/Early Pleistocene [51] and ongoing cooling since Mid-Pliocene [52] may drive the northward expansion of the hot-dry valley along river systems [43,53–55]. Deep valleys, local wind patterns, and rain shadow effects intensified bottom-valley aridity, prompting northward expansion of arid habitats, which fragmented once-continuous *A. pentaphyllum* populations and caused vicariance of lineages, especially in the southern regions. Support for this hypothesis also comes from our species distribution modeling, which revealed significant contraction during glacial period, and slightly northward expansion during warm period (Fig. 5e). Concordantly, our phylogenetic analysis found the early divergence of the southernmost populations (i.e. CDG, TK; Fig. 4), with southern populations exhibiting greater genetic differentiation compared to their northern counterparts (Fig. S15).

The weak northward gene flow observed during the Early Pleistocene (Fig. 5c) reflected a relatively warm climate compared to subsequent climate fluctuation characterized by glacial–interglacial cycles (Fig. 5a and c). After that, the species experienced continuous lineage divergence and population contraction during Early to Middle Pleistocene transition (~1.6–0.7 Mya, Fig. 5a and c), when extremely low temperatures and increased global ice volume contracted high-elevation populations (particularly northern populations). Concurrently, intensified aridity and monsoon strength in Asia during this period [56] continued southern population fragmentation. Population declines concurrent with this period have been reported in many species, including *A. yangbiense* [57], *Rhododendron griersonianum* [25], *Michelia lacei* [58], and even human ancestors, with about 98.7% lost at the beginning of this bottleneck [59]. The most recent bottleneck was detected during the Penultimate Glacial Period (~0.36–0.13 Mya), characterized by sharp reductions in population size and southward geneflow (Fig. 5a and b), potentially caused by expanding ice sheets at high elevations. Species distribution modeling revealed a significant contraction of suitable habitat since LIG (Fig. 5e), resulting in the current narrow distribution of *A. pentaphyllum*.

In addition, the significantly lower census population size (**N*_c_* ≈ 1.65 × 10^4^) investigated in the field than **N*_e_* (~1.19 × 10^6^) indicated that strong pressures have recently been imposed on *A. pentaphyllum* (Table S13; [60]). Contemporary intensifying anthropogenic activities (e.g. logging), have likely accelerated habitat fragmentation and population isolation, contributing to the current endangered status of *A. pentaphyllum*.

### Patterns of inbreeding, genetic load, and their implications for conservation strategies

Small populations are more susceptible to inbreeding depression due to their low numbers of individuals, leading to an increased probability of fixing homozygous deleterious mutations in the genome and resulting in an overall loss of diversity [61–65]. Recent studies supported that the extent of population contraction and duration of bottlenecks influenced the accumulation of deleterious mutations in small populations [66]. While seriously deleterious alleles can be effectively purged in the early stages of rapid population contraction, prolonged bottlenecking leads to their gradual accumulation and random fixation, which may further reduce the species’ fitness [67]. This demographic stress, combined with inbreeding, elevates the overall genetic load and reduces genetic variation, ultimately impairing the species’ ability to adapt to environmental changes.

In our study, the higher genetic load observed in small populations such as TKX, CDG, LM, and TES may be attributed to sustained, higher levels of inbreeding, as evidenced by the higher number of short ROH [29] and more related individual pairs (Fig. 6c, Tables S20 and S22). Furthermore, genetic isolation among adjacent populations, even within the core distribution area (e.g. cluster 6), is evident from the distinct genetic structure (Fig. S11) and varying patterns of inbreeding and mutation load across populations (Fig. 6). For example, BYP from cluster 6, the uppermost population of the valley’s tributary (Fig. 4d), exhibits high *F*_ROH_ due to its greater isolation from other populations, similar to other peripheral populations. In LTG, some of the individuals exhibit high *F*_ROH_ values, while others exhibit low *F*_ROH_ values (Table S21), causing great variance of inbreeding level in the population (Fig. 4c). Therefore, although populations in cluster 6 show relatively low *F*_ST_ among populations (Table S18), possibly due to gene flow, incomplete lineage sorting, and/or recent vicariance, some of the individuals suffered from high levels of inbreeding and need conservation attention. This phenomenon also suggests that habitat fragmentation may have resulted in numerous small, isolated populations with reduced capacity to purge deleterious mutations and decreased adaptive potential [68]. Over time, this could potentially lead to the gradual extinction of these isolated subpopulations and diminish the adaptive potential of the entire species ([21]). Functional annotation indicated that genes containing deleterious mutations were enriched in energy synthesis and transformation primarily affects processes, which encompasses nearly all biological processes of an organism. The accumulation of these deleterious mutations would potentially reduce the adaptive potential of *A. pentaphyllum* and further affect its long-term survival.

The identification of management units (MUs) and evolutionarily significant units (ESUs) is crucial for the management of natural populations. Obviously, populations TKX and CDG, located lowest reaches of the entire distribution range of *A. pentaphyllum*, exhibited the lowest nucleotide diversity (Table 1), with relatively high *F*_ROH_ and more homozygous deleterious mutations (Fig. 6). These two populations showed pure ancestry with no admixture at all values of *K* in the admixture analysis (Fig. S11), likely due to long-distance isolation from other populations (TKX vs others: ~25–129 km; CDG vs others: ~26–180 km, Table S18), which may decrease opportunities for pollen and seed dispersal. Furthermore, TKX and CDG represent two ancient diverged lineages of *A. pentaphyllum* (Fig. 4c), necessitating consideration as two separate ESUs. Similarly, LM was genetically and geographically isolated (Fig. 4d and Table S18), with low nucleotide diversity and high recent inbreeding (Fig. 6a, Table 1, and Table S20), and deserves to be treated as another ESU. The population sizes of TZC and MDG are extremely small (<50 individuals, Table S13), and their habitats have suffered significant degradation due to factors like agricultural expansion and hydropower development. Both of them should be recognized as two MUs, despite the former demonstrating a strong capacity to purge deleterious mutations (Table S28) and the latter exhibiting a low inbreeding rate (Table S20). The TES population is rather peculiar, exhibiting different genetic components from neighboring populations when *K* > 8 and forming a lineage with AJ (Fig. S11). In addition, this population is distributed on a small hillside, with few individuals, low genetic diversity (Table 1, Table S27), high inbreeding (Fig. 6c and Table S20), and a large number of homozygous DEL variants (Fig. 6e), which also should be treated as another ESU and needs further conservation action. The nearest MLC seemed to be the ideal source population for TES, due to their similar genetic background (*F*_ST_ = 0.148, Tables S18–S19) with higher genetic diversity (Table 1) and a relatively lower number of shared deleterious mutations (Table S32). We recommend introducing seedlings germinated from seeds produced by hand-cross-pollination in MLC into the TES population to increase genetic diversity in this small population.

## Conclusions

In conclusion, this study presents the first chromosome-level reference genome for *Acer pentaphyllum*, a CR maple species adapted to the dry-hot valleys of western Sichuan. Comparative genomic analyses revealed potential mechanisms underlying the species’ adaptation to its distinctive habitat, including the expansion and positive selection of genes involved in photosynthesis, plant hormone signal transduction, and plant–pathogen interaction. Our findings uncovered an unexpected upstream colonization pattern along the Yalong River and identified genetic factors contributing to the species’ endangered status. Historical climate changes, geological events, and recent anthropogenic activities have resulted in genetic bottlenecks, habitat fragmentation, and the formation of small, isolated populations with low genetic diversity. The high levels of inbreeding and genetic load observed in these populations, particularly at range edges, indicate reduced adaptive potential in the wild. This comprehensive genomic and population genetic analysis provides crucial insights for developing targeted conservation strategies, including the identification of management units and recommendations for genetic rescue. Our high-quality reference genome and extensive population data will not only contribute to the preservation of *A. pentaphyllum* but also enhance our understanding of plant adaptation to extreme environments and the impacts of climate change on species with restricted distributions.

## Materials and methods

### Plant materials and field investigations

An *ex situ* conserved individual of *A. pentaphyllum* growing at the Chengdu Institute of Biology (CIB), Chinese Academy of Sciences, was used for reference genome construction. This tree was grown from a seed in 2018, which was originally collected from Muli County, Liangshan, Sichuan. Fresh young leaves were collected for DNA extraction, library preparation, and whole genome *de novo* sequencing. Two tissues (leaves and stems) from this individual were sampled for transcriptome sequencing. Fresh young flowers were obtained from Pusharong Town, Kangding County, Sichuan in 2022. The materials were collected and immediately frozen using liquid nitrogen and then stored at −80°C for subsequent DNA and RNA extraction.

For the population material, our team conducted a comprehensive survey for all known distribution areas (including Yajiang, Kangding, Jiulong, and Muli counties) of *A. pentaphyllum* from 2021 to 2022 to estimate their census size. We recorded the number of individuals for each population found based on direct measurement methods, finally identifying 28 populations from which 227 individuals were collected and preserved using silica gel for resequencing (Table S14).

### Library preparation and sequencing

After determining the chromosome number and genome size (Appendix S1), high-quality genomic DNA was extracted from the tender leaves of *A. pentaphyllum* using a modified CTAB method [69]. To acquire an accurate genome assembly, several sequencing platforms were utilized, including Sequel II (Pacific Biosciences, Menlo Park, CA, USA) and Novaseq 6000 (Illumina, San Diego, CA, USA). For chromosome conformation capture sequencing, Hi-C libraries were constructed from tender leaves and sequenced using the MGISEQ-2000 system (BGI, Shenzhen, China). RNA was extracted from different tissues to create cDNA libraries using TRIzol reagent (Invitrogen, Carlsbad, CA, United States). The libraries were further purified and quality control (QC) to obtain Iso-Seq SMRT bell libraries, which were sequenced using Novaseq 6000. Raw sequencing data were filtered with fastp v0.23.2 [70] to remove low-quality reads, adapter contamination, and PCR duplicates before further analysis.

### *De novo* assembly, assessment, and annotation of reference genome

In this study, we employed a strategy of ‘assembling primarily with third-generation data and error correction with second-generation data’ to assemble the genome of *A. pentaphyllum*. Initially, HiFiasm v0.16.1 [71] software was used to assemble filtered PacBio long reads with the parameter ‘—hom-cov 46’, yielding a primary assembly. The primary assembly was then polished with the Illumina short reads using Pilon v1.24 [72] for three iterations to obtain a draft genome of *A. pentaphyllum*. Finally, Hi-C reads were aligned to the draft genome using BWA-MEM v0.7.17 [73], and contigs were clustered at the chromosome level using Lachesis [74] based on interaction information recorded in the data, generating the chromosome-level reference genome of *A. pentaphyllum*.

To assess the completeness and consistency of the reference genome of *A. pentaphyllum*, we employed Minimap v2.1 [75] to align the Illumina short reads to the genome, and SAMTools v1.6 (−flagstat; [76]) was used to statistics the mapping rate, coverage, and average sequencing depth. Concurrently, the Benchmarking Universal Single-Copy Orthologs5 (BUSCO5) test was performed to evaluate the quality of the assembly using the Eudicots_odb10 database (https://buscodata.ezlab.org/v5/data/lineages/), which contains 2326 conserved eukaryotic genes [77].

The genomic components, including repetitive elements, gene structures, and non-coding RNAs (ncRNA), were annotated through a combination of *ab initio* prediction, homology-based alignment, and transcripts from different tissues (see details in Appendix S2).

### Comparative genome analysis

We performed comparative genomic analyses using the genomic sequences of *A. pentaphyllum* and 14 related published plant species: *A. yangbiense*, *A. truncatum, A. catalpifolium*, *A. saccharum, A. negundo*, *A. pseudosieboldianum*, *Dipteronia dyeriana*, *Dipteronia sinensis* [21], *Arabidopsis thaliana* [78], *C. sinensis* [79], *Eucalyptus grandis* [80], *Nephelium lappaceum* [81], *V. vinifera* [82], and *Xanthoceras sorbifolium* [14]. OrthoFinder v2.5.4 [83] was used to group orthologous and paralogous genes among the 15 genomes based on a Markov Cluster Algorithm (MCL). A total of 1422 single-copy orthologous genes were shared by all 15 species and used to construct a maximum likelihood tree using RAxML [84] and IQ-TREE v2 [85] software. The specific genes of *A. pentaphyllum* were annotated and functionally enriched by GO and KEGG analyses.

To estimate the divergence time between *Acer* plants, the MCMCtree program in PAML [86] was utilized, with four corrected divergence time points from the TimeTree website (http://www.timetree.org/): *A. yangbiense* vs. *C. sinensis* (95% highest posterior density, HPD: 68–82.8 Mya), *A. yangbiense* vs. *A. thaliana* (95% HPD: 90–99.9 Mya), *A. yangbiense* vs. *E. grandis* (95% HPD: 100–128 Mya), and *A. yangbiense* vs. *V. vinifera* (95% HPD: 109–123.5 Mya), and one corrected divergence time point from the fossil records [21,87]: *Acer* vs. *Dipteronia* (56–73 Mya, based on the earliest fossil records of the genus *Dipteronia* dating back to 63 Mya).

Based on the result of gene family clustering and ultrametric tree containing the divergence times, CAFÉ [88] and CODEML [86] programs were used to calculate the number of gene families undergoing expansion, contraction, and positive selection. Genes that exhibited significant expansion, contraction, and strong positive selection in the *A. pentaphyllum* genome were further annotated and functionally enriched by GO and KEGG analyses (see details in Appendix S3).

### Inference of WGD events and LTR insertions

WGD events are a ubiquitous phenomenon in plants (especially in angiosperms), contributing to variations in both the size and structure of the genome and providing an important driver for species evolution and speciation. We conducted all-vs-all BLAST (*E*-value = 1e-5) on the protein sequences of *A. pentaphyllum*, *A. yangbiense*, *A. pseudosieboldianum*, and *Dipteronia sinensis*. Next, JCVI v1.2.7 [89] software was employed to perform collinearity analyses among the three Sapindaceae species with the sweet orange and grape genomes as references. Additionally, WGDI v0.5.9 [90] was utilized to calculate the synonymous substitution rates (**K*_s_*) of collinear genes, aiming to infer potential WGD events in the *Acer pentaphyllum* genome.

To infer the history of TE activity and selective pressures against LTR-RTs in the *A. pentaphyllum* genome, we calculated the insertion time for each of the LTR-RTs. Firstly, the mutation rate ($\mu$) of *A. pentaphyllum* was estimated using the formula $\mu ={K}_S/2T$, where ${K}_S$ represents the synonymous substitution rate peak value for all paralogous genes and $T$ is the estimated divergence time between two species that had been evaluated in the phylogenomic analyses outlined above, and thus $\mu$ was calculated to be 4.66 × 10^−9^ per site per year. Secondly, LTR pairs from the results of LTRharvest and LTR_FINDER were aligned using MUSCLE v5 [91]. After trimming gaps on the ends of aligned sequences, we assessed the distance between the two LTR-RTs for all LTR pairs, and the insertion times could be calculated using the inverse formula $T={K}_S/2\mu$ [92,93].

### Genome mapping and SNP calling

The original resequencing data, including 227 individuals of *A. pentaphyllum* and 2 closely related species: *A. yangbiense* (PRJNA524417) and *D. sinensis* (PRJNA796066.), underwent filtering using fastp to discard the adaptors, duplicates, and low-quality sequences, with parameters of ‘--length_required 50 --cut_window_size 4 --cut_mean_quality 15’. After quality control, paired-end clean data were mapped to the reference genome of *A. pentaphyllum* using BWA-MEM [73]. SAMTools [76] was then used to filter out the unaligned and low-quality reads. Only the bases with a quality score ≥ 20 and reads with a mapping quality score ≥ 20 were considered in the subsequent analyses. The MarkDuplicates module in Picard v1.1 (http://broadinstitute.github.io/picard/) was employed to mark and remove the duplicate reads generated during the sequencing process with default parameters.

The Genome Analysis Toolkit (GATK) pipeline v4.2.0.0 [94] was performed for variants calling and preliminary filtering with parameters of ‘QD < 2.0, MQ < 40.0, FS > 60.0, SOR > 3.0, MQRankSum < -12.5, ReadPosRankSum < -8.0’, obtaining a total of 4 775 335 high-quality SNPs (Dataset 1) with an average of 21 036 SNPs per individual. The resulting dataset was further filtered by utilizing VCFtools v0.1.15 [95] software, involving the following steps: (i) removal of SNPs with a quality score < 30 (−-minQ 30); (ii) removal of non-bi-allelic SNPs and non-SNPs (−-max-alleles 2 --min-alleles 2); (iii) removal of SNPs with a depth ≥ 2 times or ≤ 1/3 of the average sequencing depth (−-maxDP 47 --minDP 8); and (4) removal of SNPs with a missing rate > 10% (−-max-missing 0.9), generating 3 818 766 SNPs (Dataset 2). Furthermore, SNPs with minor allele frequency lower than 0.01 (−-maf 0.01) in Dataset 2 were removed, and the remaining loci (2 757 997, Dataset 3) were used for downstream analysis. In addition, variable sites in Dataset 3 were annotated using ANNOVAR [96] software.

### Population genetic diversity and estimation of inbreeding

Linkage disequilibrium (LD) decay analysis was performed across the *A. pentaphyllum* genome using PopLDdecay v3.42 [97] software, which allows us to obtain LD statistics directly from SNP Dataset1. Different population genetic estimators, including nucleotide diversity (*π*), Tajima’s *D*, and fixation index of subdivision (*F*_ST_), were calculated using VCFtools with a sliding window size of 100 Kb.

To assess the inbreeding level among populations, based on SNP Dataset 2 generated previously, we first calculate the kinship coefficient between individuals of *A. pentaphyllum* to identify potential relatives or clones using a robust relationship inference algorithm KING v2.3.2 [98]. VCFtools (−-het) was then used to calculate each individual’s inbreeding coefficients (*F*_IS_) based on their heterozygosity. Furthermore, we estimated the runs of homozygosity (ROHs) for each sampled tree using PLINK software with parameters of ‘--homozyg-density 10 --homozyg-gap 100 --homozyg-kb 100 --homozyg-snp 10’. We assessed the fraction of ROHs on the genome (${F}_{ROH}$) using the formula ${F}_{ROH}=\frac{\sum{L}_{ROH}}{L_{auto}}$, where $\sum{L}_{ROH}$ is the total length of ROHs that are longer than 100 Kb and have at least 10 SNPs in the genome and ${L}_{auto}$ is the total length of the *A. pentaphyllum* reference genome. ${F}_{ROH}$ was calculated and were visualized via Manhattan plots using the qqman v0.1.9 (https://cran.rproject.org/web/packages/qq-man/) package.

### Population structure

PLINK v1.9 [99] was used to filter out linked sites in Dataset 3 with the parameter of ‘--indep-pairwise 50 10 0.2’. After filtering, a total of 548 609 loci (Dataset 4) were obtained for inferring the population genetic structure of *A. pentaphyllum* using ADMIXTURE v1.3 [100] software. The number of assumed ancestral populations (*K*) varied from 2 to 20 and the optimal *K* value was determined by the lowest CV error. Then, GCTA v1.93.2 [101] was employed for performing principal component analysis (PCA) to detect population stratification and evolutionary relationships. Furthermore, we reconstructed an ML phylogenetic tree using RAxML with *A. yangbiense* and *D. sinensis* as outgroups.

### Lineage divergence and inference of demographic history

We employed the pairwise sequentially Markovian coalescent (PSMC) method [102] to reconstruct historical changes in effective population size (*Ne*) based on representative individuals for each cluster and population. The mutation rate was set as 4.66 × 10^−8^ per site per 10 years based on the previous calculation and the generation time of *A. pentaphyllum* was set as 10 years according to field observations (from seed to seed). To recover the demographic history and explore the role of gene flow in the process of speciation and lineage divergence of *A. pentaphyllum*, we conducted composite maximum likelihood (ML) inference in FASTSIMCOAL2 v2.6 [30] as complementary based on site frequency spectrum (SFS, see Appendix S4 for details). Stairway Plot 2 [103] was also used to infer changes in population size through time based on the unfolded SFS of each group. In addition, we estimated the *Ne* of *A. pentaphyllum* using the inverse formula *N_e_* = *θ/(4 μ)*, where *θ* represents the expected genetic diversity (or heterozygosity) in a population calculated using: $\theta =S/{a}_n$. S is the proportion of segregating sites in the sample: $S={N}_{SNP}/{L}_{genome}$ and ${a}_n$ is defined as the sum of the inverses of the sample size (*n*): ${a}_n=\sum_{i=1}^{n-1}1/i$. *μ* represents the mutation rate per site per year [104].

To further explore the effects of paleoclimate changes on the dispersal patterns and demographic history of *A. pentaphyllum*, we simulated the species’ optimal distribution areas using 175 non-redundant collection sites (Table S31) for different periods, including LIG, LGM, current, and 2080–2100 under shared socioeconomic pathways (SSPs) 126 periods (methods see details in Appendix S5).

### Estimation of genetic load and deleterious mutations

To assess the potential influence of genetic load in *A. pentaphyllum*, we employed three different methods to detect deleterious mutations using polarized SNPs (filtering out sites where reference alleles inconsistent with ancestral states). We first calculated the Grantham distance [105] for each nonsynonymous SNP, and radical missense substitution (RADICAL) was identified when the Grantham score > 150. SIFT4G v2.0.0117 [106] software was then performed to annotate the variant sites from vcf files using SIFT4G_Annotator.jar (https://github.com/pauline-ng/SIFT4G_Annotator). SNPs with a score < 0.05 were assumed to be deleterious (DEL). In addition, we searched through the whole genome to predict severely deleterious mutations that caused start loss, stop gain, stop loss, and changes in splice sites with the help of annotation information. To further access the accumulation of severely deleterious mutations among the different populations and groups of *A. pentaphyllum*, SNPEFF v4.5 [107] was used to annotate SNPs leading to gene loss of function (LOF) with the parameter ‘-lof’.

Additionally, to assess the purging efficiency of deleterious mutations among different populations and clusters of *A. pentaphyllum,* a modified *R*_X/Y_ method [66,108] from Feng [109] was applied. We first calculated the mean ratio of derived alleles ($p$) for each population using$p=\sum_{i=1}^n\frac{N_{derived\ alleles}^{class}}{N_{SNPs}^{total}\times 2}/n$ for all types of deleterious mutations, where ${N}_{derived\ alleles}^{class}$ is the number of derived allelic genes for three types of mutations (DEL, RADICAL, and LOF), which is based on counting each heterozygous site once and each homozygous derived site twice for each individual, ${N}_{SNPs}^{total}$ is the total number of SNPs used for deleterious mutations annotation, and $n$ represents the number of individuals in a population of *A. pentaphyllum*. The mean ratio of derived alleles for all SNPs (${p}^{total}$) was used for correction, and the relative ratio of mutant alleles (${R}_p$) was calculated as ${R}_p={p}^{class}/{p}^{total}$ to account for inconsistencies in derived allele accumulation across different clusters. Here, ${p}^{class}$ is the average derived alleles ratio for a specific type of harmful mutations, and ${p}^{total}$ is the average derived allele ratio of the total number of SNPs. A lower relative ratio indicates a stronger purging efficiency of deleterious mutations.

## Supplementary Material

Web_Material_uhae357

## Data Availability

All data that support the findings of this study, including sequencing data, reference genome, and gene annotations, have been deposited into CNGB Sequence Archive (CNSA; [110]) of China National GeneBank DataBase (CNGBdb) with accession number CNP0006021 (reviewer link: http://db.cngb.org/cnsa/project/CNP0006021_715ecb62/reviewlink/).
