# Supplementary material for: High-resolution genome assembly and population genetic study of the endangered maple Acer pentaphyllum (Sapindaceae): implications for conservation strategies
Source: Hortic Res. 2024 Dec 17;12(4):uhae357. doi: 10.1093/hr/uhae357 (PMC11891484; doi:10.1093/hr/uhae357)
Supplement: Web_Material_uhae357 [file web_material_uhae357.zip › Supplementary methods and figures_clean.pdf]

Supplementary methods

Appendix S1. Determination of chromosome number and estimation of genome features

To observe the chromosome number in *Acer pentaphyllum*, root tips from young seedlings are collected and pretreated with 8-hydroxyquinoline to arrest cells in metaphase. After a brief rinse, they are fixed in an ethanol-acetic acid solution and hydrolyzed in hydrochloric acid at 60°C, which softens the tissue for easier staining and visualization. The root tips are then stained using aceto-orcein or Giemsa to highlight the chromosomes. After staining, the sample is placed on a slide with acetic acid and squashed under a coverslip, spreading the cells for clear viewing.

We determined the genome size and C-value (chromatin-value) using the DNA flow cytometry method (Doležel *et al.*, 2007). By comparing the fluorescence peak value of *A. pentaphyllum* with an internal control species (*Oryza Sativa* ssp. *japonica*, ~430 Mb), the genome size can be calculated using the formula: DNA size of sample = DNA size of internal control species × (fluorescence intensity of sample/fluorescence intensity of internal control species). Also, the k-mer analysis (Marcais and Kingsford, 2011) was used to estimate the genome characteristics of *A. pentaphyllum* using the data from Illumine sequencing. First, to acquire high-quality sequences, we filtered out all low-quality reads with adapters, 20% bases having Phred quality < 5 and > 10% N content using fastp v0.23.2 (Chen *et al.*, 2018). Then, jellyfish v2.3.0 (Marcais and Kingsford, 2011) was used to calculate the number and depth of k-mers with the parameter '-m 17'. Finally, GenomeScope (Vurture *et al.*, 2017) was used to estimate

the genome size and other features, including repeat contents, GC contents, and heterozygosity.

Appendix S2. Genome annotation

Repeat annotation

We employed two strategies to annotate repeat sequences across the *A. pentaphyllum* genome: homology-based and *de novo* prediction. Firstly, MITE-Hunter software (Han and Wessler, 2010) was utilized to identify the Miniature Inverted Repeat Transposable Elements (MITEs), which belong to Class II transposable elements (TEs) and are widely distributed throughout the genome. Secondly, LTRharvest (Ellinghaus *et al.*, 2008) and LTR_FINDER (Xu and Wang, 2007) were employed for identifying the highest proportion of long terminal repeat-retrotransposons (LTR-RTs) in the *A. pentaphyllum* genome, and LTR_retriever (Ou and Jiang, 2018) was then used to integrate the predictions from two software, constructing a high-quality LTR library. In addition, the assembly was compared with the Repbase database (https://www.girinst.org/repbase/) using RepeatMasker (Tarailo-Graovac and Chen, 2009) to search for consensus sequences. Finally, repeat sequences were identified and classified *de novo* using RepeatModeler2 (Flynn *et al.*, 2020). The above results were further integrated to perform repeat prediction for *A. pentaphyllum*.

Gene structure prediction

Transcriptome sequences, homologous proteins, and *de novo* predictions were utilized to estimate the gene structure of the genome, involving the following steps: (1)

The transcriptome sequence data from different organs of *A. pentaphyllum* were processed using HISAT2 (Kim *et al.*, 2019). The generated full-length transcripts were then aligned to the genome using PASA (Haas *et al.*, 2003), through which corresponding prediction open reading frame (ORF) models were obtained; (2) 140,473 nonredundant protein sequence data from closely related species, including *A. yangbiense*, *A. pseudosieboldianum*, *A. truncatum*, *A. catalpifolium*, and *Sapindus mukorossi* Gaertn, were compared with the reference genome of *A. pentaphyllum* using GeMoMa (Keilwagen *et al.*, 2019). Subsequently, the alignment results from all homologous species were combined to determine the exon and intron boundaries for the predicted genes; (3) For *de novo* estimation based on the genes assembled from the transcriptome data, reliable genes were chosen to train models using AUGUSTUS (Stanke *et al.*, 2004), SNAP (Korf, 2004), GlimmerHMM (Majoros *et al.*, 2004), and GeneMark-ET (Hoff *et al.*, 2016) software to predict gene structure in the genome, obtaining an *A. pentaphyllum* prediction model. Following the integrated gene prediction results obtained by the three methods above using the EvidenceModeler (Haas *et al.*, 2008), the initial prediction genomic gene set of *A. pentaphyllum* was obtained. Finally, UTR regions and alternative splicing on the genome were annotated using PASA based on the comparison of transcripts with the predicted genes, after which the final gene set was obtained for subsequent analysis.

Non-coding RNA annotation

Non-coding RNAs (ncRNAs) are functional RNA molecules that cannot be translated into proteins, including tRNAs, rRNAs, and small RNAs such as microRNAs,

siRNAs, and so on. Compared to traditional translatable mRNA, ncRNAs are shorter and less abundance within cells but play crucial roles in t gene expression regulation. tRNAscan-SE v2.0 (Lowe and Eddy, 1997) was employed to *de novo* predict tRNAs in the genome of *A. pentaphyllum*, while RNAmmer (Lagesen *et al.*, 2007) was used to predict the rRNA and subunits. Additionally, the reference genome was compared with the Rfam database (http://rfam.xfam.org/) using Infernal software (Nawrocki and Eddy, 2013) to annotate other non-coding RNA. The above results were further integrated to perform ncRNA prediction for *A. pentaphyllum*.

Gene function annotation

Gene function annotation was performed for five public protein databases via BLASTP (https://blast.ncbi.nlm.nih.gov/Blast.cgi?PROGRAM=blastp), including Non-Reduntant Protein Database (NR, https://ftp.ncbi.nlm.nih.gov/blast/db/FASTA/), Gene Ontology (GO, http://geneontology.org/), Kyoto Encyclopedia of Gene and Genomes (KEGG, https://www.kegg.jp/), evolutionary genealogy of genes: Non-supervised Orthologous Groups 5.0 (eggNOG, http://eggnog5.embl.de/download/eggnog5.0/), and Swissport (https://www.uniprot.org/).

Appendix S3. Positive selection analysis

PAL2NAL (Suyama *et al.*, 2006) was employed to convert single-copy orthologous protein sequences of 15 species into codon-based alignment CDS sequences, serving as input files for the CODEML program in PAML (Yang, 2007). The specific steps of positive selection analysis are as follows: the null hypothesis

analysis was initiated by setting fix_omega = 1 and omega = 1, yielding likelihood value l0, then the alte hypothesis analysis was repeated by setting fix_omega = 0 and omega = 1.5, yielding l1. After conducting a chi-square test using the command "chi2 1 2Δ(l1-l0)", genes with p-value <0.05 were considered under positive selection. GO functional annotations were performed on these genes to infer their potential functions.

Appendix S4. Lineage divergence and inference of demographic history

To infer lineage divergence times within *A. pentaphyllum,* we extracted 225 single-copy orthologous genes from five representative individuals and dated the phylogeny including six *Acer* species and two *Dipteornia* species using MCMCTREE (Yang, 2007) with the same calibrations used in the comparative genome analysis. These inferred divergence times were used to set the prior distribution of divergence in the FASTSIMCOAL2 (Excoffier *et al*., 2013) simulations.

In addition, to recover the demographic history and explore the role of gene flow in the process of speciation and lineage divergence of *A. pentaphyllum*, we conducted composite maximum likelihood (ML) inference as complementary based on site frequency spectrum (SFS). To generate the unfolded site frequency spectrum (u-SFS), we inferred the probability of the derived versus ancestral allelic state using est-sfs v2.03 (Pickrell and Pritchard, 2012). One individual of *D. sinensis* and *A. yangbiense* was used as the outgroups in the est-sfs analysis, leaving 679,558 ancestral state sites (Polarized SNPs). The joint unfolded 2D-SFS were built using neutral SNPs with using easySFS.py (https:// github.com/isaacovercast/easySFS). The SNP data of each cluster

were down-projected to an SFS with relatively similar sampling sizes (18 and 20) across groups to decrease the effect of different levels of missing data between clusters.

Eight representative scenarios were set focusing on changes in population sizes and occurrence and magnitude of gene flow among populations grouped based on genetic structure and geographical distributions (i.e., TK, CDG, ML, JL, KD) of *A. pentaphyllum* (Figure S17). We calculated the likelihood function for different demographic scenarios using the software FASTSIMCOAL2 v2.6 (Excoffier et al. 2013). For each scenario, 100,000 coalescent simulations per likelihood estimation (-n 100,000) and 40 expectation-conditional maximization (ECM) cycles (-L40) were used as the command line parameters for each run. The Akaike information criterion (AIC) was used to compare different models. In this case, AIC = 2k–2ln(MaxEstLhood), where k is the number of parameters estimated by each model, and MaxEstLhood is the ML function value for each model. Moreover, when searching for a maximum likelihood value, FASTSIMCOAL2 may reach a local optimum instead of a global optimum. Thus, we repeated each step at least twice (results not shown), to ensure we were not ending in a local optimum, thereby getting better estimates of the global optimum.

Appendix S5. Species distribution modeling of past, current, and future

To further explore the effects of paleoclimate changes on the dispersal patterns and demographic history of *A. pentaphyllum*, we simulated the species' optimal distribution areas for different periods. Firstly, climatic data including Last Interglacial (LIG), Last

Glacial Maximum (LGM), current, and 2080-2100 under shared socioeconomic pathways (SSPs) 126 periods were downloaded from the WorldClim website (https://worldclim.org/) at a spatial resolution of 30 seconds (~900 m at the equator). Based on present climate data, ENMTools v1.0 (Warren *et al*., 2021) was used to remove redundant sites within each raster from the total 3,892 *A. pentaphyllum* collection sites, resulting in 175 distinct sites (Table S31) with an average of 6.25 sites per population. Then, 19 bioclimatic variables data of these filtered sites from different periods were extracted using ArcGIS v10.7, and subsequently analyzed for Pearson's correlation. Variables with correlation values $|r| \leq 0.8$ were retained, while pairs of variables with $|r| > 0.8$ were reduced to a single representative. Finally, only 7 variables were retained for use in the MaxEnt model predictions, specifically: bio2 (Mean Diurnal Range), bio3 (Isothermality), bio4 (Temperature Seasonality), bio6 (Min Temperature of Coldest Month), bio7 (Temperature Annual Range), bio14 (Precipitation of Driest Month), and bio18 (Precipitation of Warmest Quarter).

Based on the above selected 175 sites and 7 bioclimatic variables, MaxEnt v3.4.1 (Phillips *et al*., 2006) was utilized to model the potential distribution of *A. pentaphyllum* across 4 different periods. Bootstrap resampling was performed 10 times, with a maximum of 5,000 iterations. The potential distribution was then constructed by averaging the results of 25 simulation runs and was further classified in ArcGIS by habitat suitability into four categories: low (0–0.18), moderate (0.18–0.39), high (0.39–0.74), and very high suitability (0.74–0.99) zones.

References

Chen, S.F., Zhou, Y.Q., Chen, Y.R. and Gu, J. (2018) fastp: an ultra-fast all-in-one FASTQ preprocessor. *Bioinformatics*, **34**, i884-i890.

Doležel, J., Greilhuber, J. and Suda, J. (2007) Flow cytometry with plants: an overview. *Flow cytometry with plant cells: analysis of genes, chromosomes and genomes*, 41-65.

Ellinghaus, D., Kurtz, S. and Willhoeft, U. (2008) LTRharvest, an efficient and flexible software for de novo detection of LTR retrotransposons. *BMC Bioinformatics*, **9**, 18.

Excoffier, L., Dupanloup, I., Huerta-Sánchez, E., Sousa, V.C. and Foll, M. (2013) Robust Demographic Inference from Genomic and SNP Data. *PLOS Genetics*, **9**.

Flynn, J.M., Hubley, R., Goubert, C., Rosen, J., Clark, A.G., Feschotte, C. and Smit, A.F. (2020) RepeatModeler2 for automated genomic discovery of transposable element families. *Proceedings of the National Academy of Sciences of the United States of America*, **117**, 9451-9457.

Han, Y. and Wessler, S.R. (2010) MITE-Hunter: a program for discovering miniature inverted-repeat transposable elements from genomic sequences. *Nucleic Acids Research*, **38**, e199.

Haas, B.J., Delcher, A.L., Mount, S.M., Wortman, J.R., Smith, R.K., Hannick, L.I., Maiti, R., Ronning, C.M., Rusch, D.B., Town, C.D., Salzberg, S.L. and White, O. (2003) Improving the genome annotation using maximal transcript

alignment assemblies. *Nucleic Acids Research*, **31**, 5654-5666.

Haas, B.J., Salzberg, S.L., Zhu, W., Pertea, M., Allen, J.E., Orvis, J., White, O., Buell, C.R. and Wortman, J.R. (2008) Automated eukaryotic gene structure annotation using EVidenceModeler and the program to assemble spliced alignments. *Genome Biology*, **9**.

Hoff, K.J., Lange, S., Lomsadze, A., Borodovsky, M. and Stanke, M. (2016) BRAKER1: Unsupervised RNA-Seq-Based Genome Annotation with GeneMark-ET and AUGUSTUS. *Bioinformatics*, **32**, 767-769.

Keilwagen, J., Hartung, F. and Grau, J. (2019) GeMoMa: Homology-Based Gene Prediction Utilizing Intron Position Conservation and RNA-seq Data. *Methods in Molecular Biology*. 1962, 161-177.

Kim, D., Paggi, J.M., Park, C., Bennett, C. and Salzberg, S.L. (2019) Graph-based genome alignment and genotyping with HISAT2 and HISAT-genotype. *Nature Biotechnology*, **37**, 907-+.

Korf, I. (2004) Gene finding in novel genomes. *Bmc Bioinformatics*, **5**.

Lagesen, K., Hallin, P., Rodland, E.A., Stærfeldt, H.H., Rognes, T. and Ussery, D.W. (2007) RNAmmer: consistent and rapid annotation of ribosomal RNA genes. *Nucleic Acids Research*, **35**, 3100-3108.

Lowe, T.M. and Eddy, S.R. (1997) tRNAscan-SE: A program for improved detection of transfer RNA genes in genomic sequence. *Nucleic Acids Research*, **25**, 955-964.

Marcais, G. and Kingsford, C. (2011) A fast, lock-free approach for efficient parallel

counting of occurrences of k-mers. *Bioinformatics*, **27**, 764-770.

Majoros, W.H., Pertea, M. and Salzberg, S.L. (2004) TigrScan and GlimmerHMM: two open source eukaryotic gene-finders. *Bioinformatics*, **20**, 2878-2879.

Nawrocki, E.P. and Eddy, S.R. (2013) Infernal 1.1: 100-fold faster RNA homology searches. *Bioinformatics*, **29**, 2933-2935.

Ou, S. and Jiang, N. (2018) LTR_retriever: A Highly Accurate and Sensitive Program for Identification of Long Terminal Repeat Retrotransposons. *Plant Physiology*, **176**, 1410-1422.

Phillips, S.J., Anderson, R.P. and Schapire, R.E. (2006) Maximum entropy modeling of species geographic distributions. *Ecological Modelling*, **190**, 231-259.

Pickrell, J.K. and Pritchard, J.K. (2012) Inference of population splits and mixtures from genome-wide allele frequency data. *PLOS Genetics*, **8**, e1002967.

Stanke, M., Steinkamp, R., Waack, S. and Morgenstern, B. (2004) AUGUSTUS: a web server for gene finding in eukaryotes. *Nucleic Acids Research*, **32**, W309-W312.

Suyama, M., Torrents, D. and Bork, P. (2006) PAL2NAL: robust conversion of protein sequence alignments into the corresponding codon alignments. *Nucleic Acids Research*, **34**, W609-W612.

Tarailo-Graovac, M. and Chen, N. (2009) Using RepeatMasker to Identify Repetitive Elements in Genomic Sequences. *Curr Protoc Bioinformatics*, **25**, Chapter 4:Unit 4.10.

Vurture, G.W., Sedlazeck, F.J., Nattestad, M., Underwood, C.J., Fang, H.,

Gurtowski, J. and Schatz, M.C. (2017) GenomeScope: fast reference-free genome profiling from short reads. *Bioinformatics*, **33**, 2202-2204.

Warren, D. L., Matzke, N. J., Cardillo, M., Baumgartner, J. B., Beaumont, L., Turelli, M., Glor, R. E., Huron, N. A., Simões, M., Iglesias, T. L., Piquet, J. C. and Dinnage, R. (2021). ENMTools 1.0: an R package for comparative ecological biogeography. *Ecography*, **44**, 504-511.

Xu, Z. and Wang, H. (2007) LTR_FINDER: an efficient tool for the prediction of full-length LTR retrotransposons. *Nucleic Acids Research*, **35**, W265-268.

Yang, Z. (2007) PAML 4: phylogenetic analysis by maximum likelihood. *Molecular Biology and Evolution*, **24**, 1586-1591.

Supplementary figures

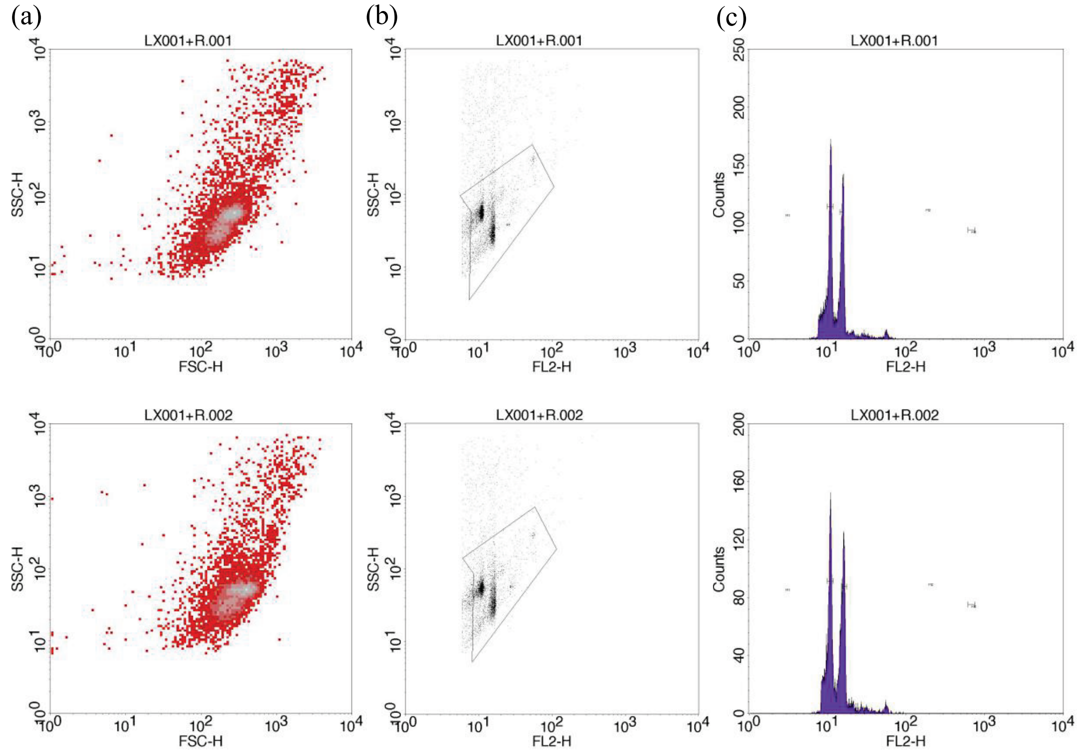

Figure S1. Flow cytometry analysis of *A. pentaphyllum.* (a) Scatter plot of side scatter (SSC-H) versus forward scatter (FSC-H). (b) The gating strategy based on side scatter (SSC-H) versus fluorescence intensity (FL2-H). (c) Histogram of fluorescence intensity (FL2-H).

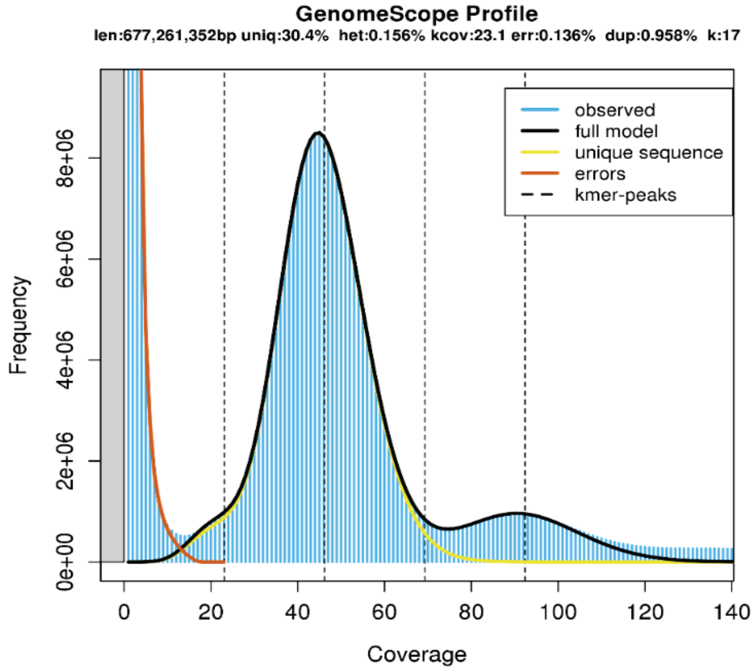

Figure S2. Estimation of genome features of *A. pentaphyllum* based on 17-mer analysis.

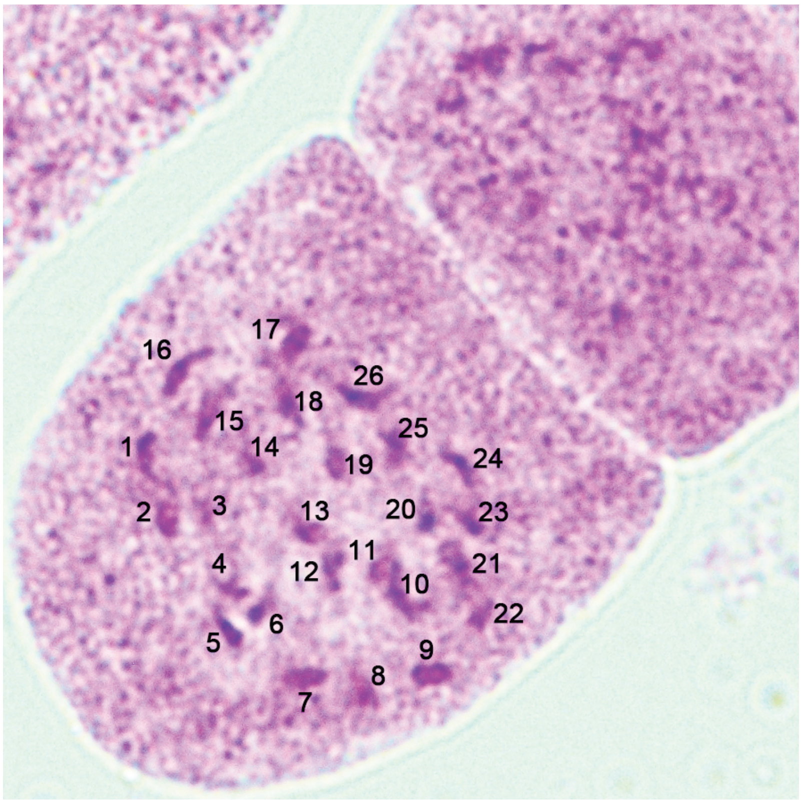

Figure S3. Chromosome squashing image of root tip tissue from *A. pentaphyllum* seedlings.

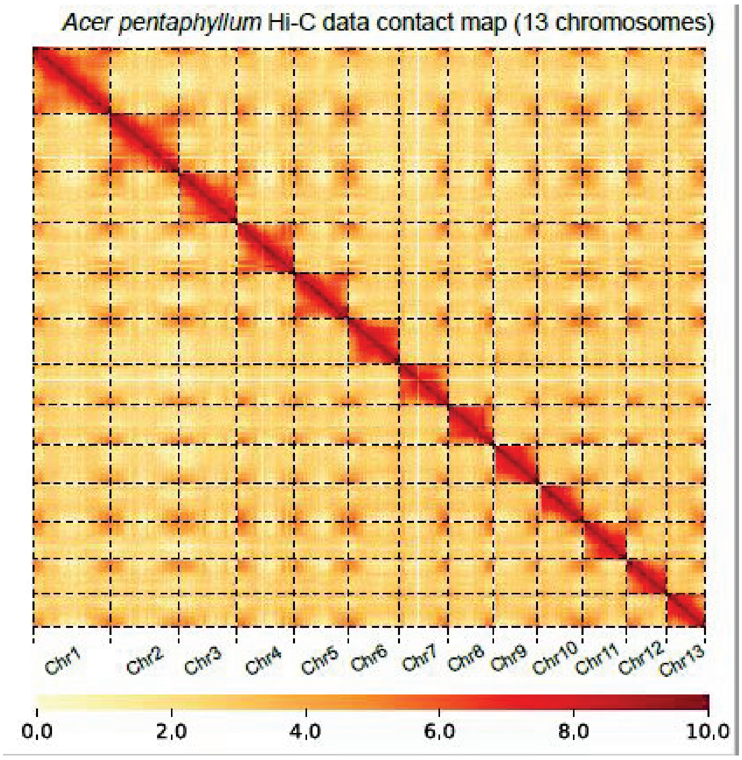

Figure S4. Hi-C intrachromosomal contact map of 13 chromosomes for *A. pentaphyllum.*

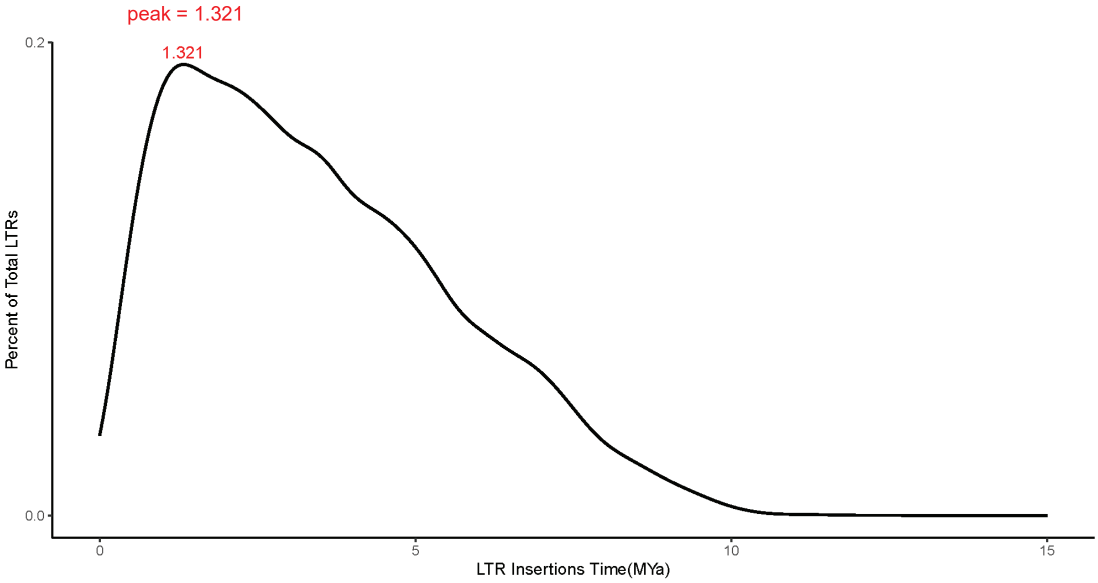

Figure S5. Distribution of LTR transposons retrotransposons insertion times on the *A. pentaphyllum* genome *A. pentaphyllum.*

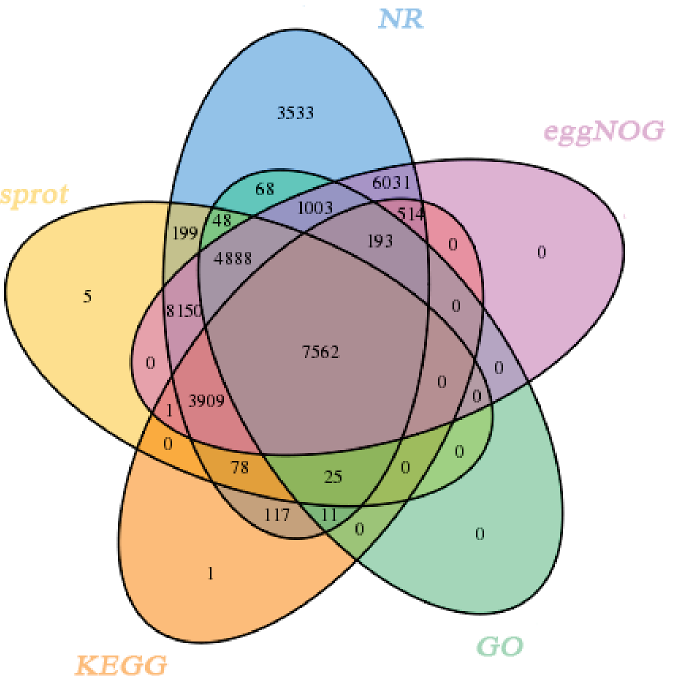

Figure S6. Venn diagram of functional annotation for *A. pentaphyllum* based on 5 databases.

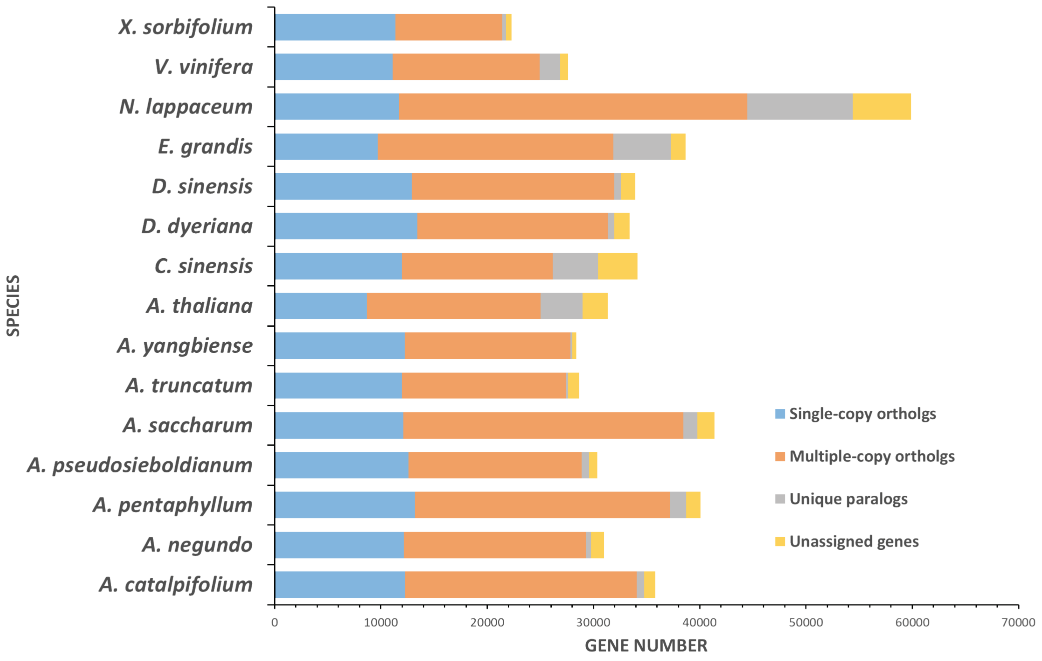

Figure S7. Number of genes in different types of gene families among 15 woody species.

(a)

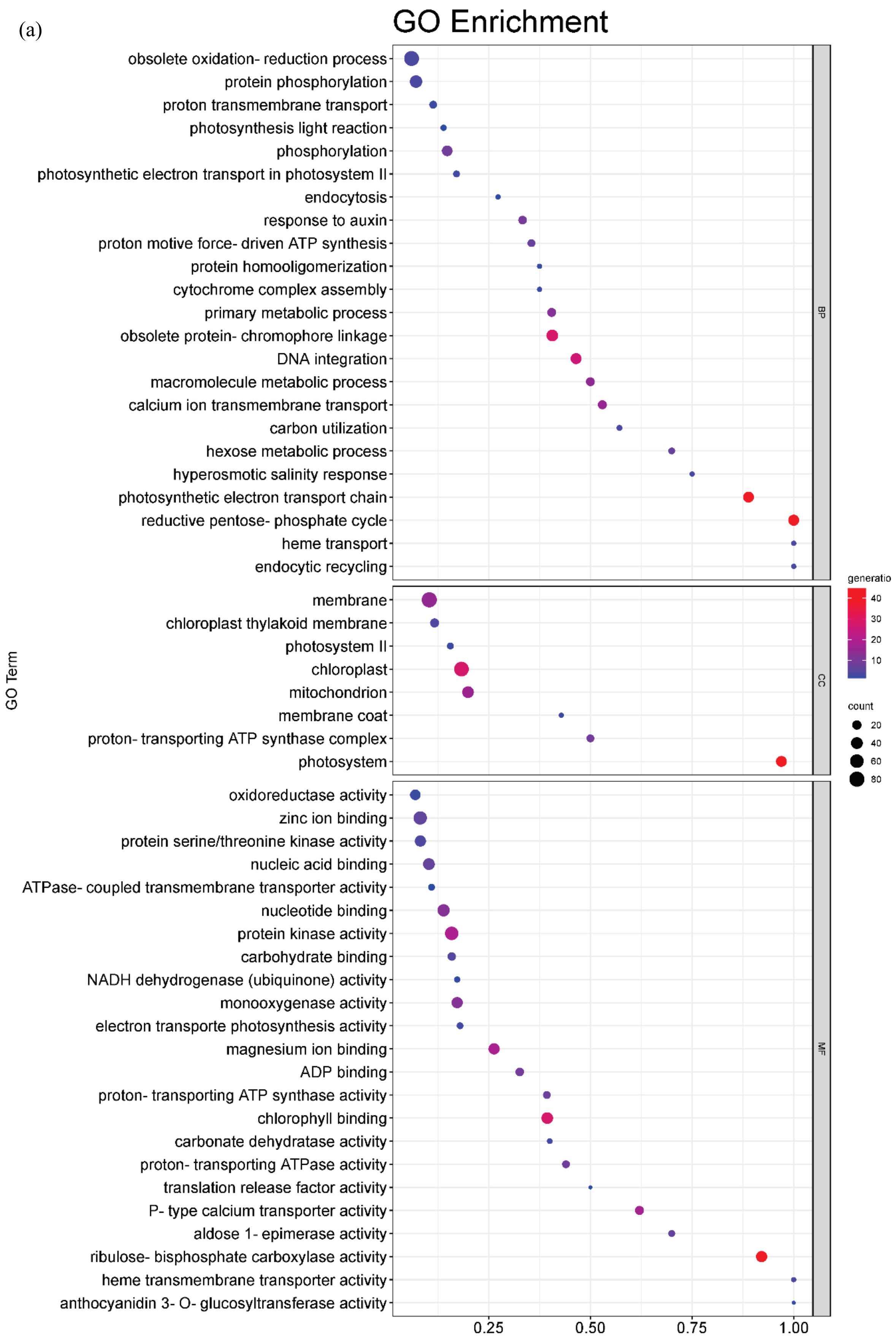
GO Enrichment
obsolete oxidation- reduction process
protein phosphorylation
proton transmembrane transport
photosynthesis light reaction
phosphorylation
photosynthetic electron transport in photosystem II
endocytosis
response to auxin
proton motive force- driven ATP synthesis
protein homooligomerization
cytochrome complex assembly
primary metabolic process
obsolete protein- chromophore linkage
DNA integration
macromolecule metabolic process
calcium ion transmembrane transport
carbon utilization
hexose metabolic process
hyperosmotic salinity response
photosynthetic electron transport chain
reductive pentose- phosphate cycle
heme transport
endocytic recycling
membrane
chloroplast thylakoid membrane
photosystem II
chloroplast
mitochondrion
membrane coat
proton- transporting ATP synthase complex
photosystem
oxidoreductase activity
zinc ion binding
protein serine/threonine kinase activity
nucleic acid binding
ATPase- coupled transmembrane transporter activity
nucleotide binding
protein kinase activity
carbohydrate binding
NADH dehydrogenase (ubiquinone) activity
monooxygenase activity
electron transporte photosynthesis activity
magnesium ion binding
ADP binding
proton- transporting ATP synthase activity
chlorophyll binding
carbonate dehydratase activity
proton- transporting ATPase activity
translation release factor activity
P- type calcium transporter activity
aldose 1- epimerase activity
ribulose- bisphosphate carboxylase activity
heme transmembrane transporter activity
anthocyanidin 3- O- glucosyltransferase activity
GO Term
BP
CC
MF
0.25
0.50
0.75
1.00
generatio
40
30
20
10
count
20
40
60
80

(b)

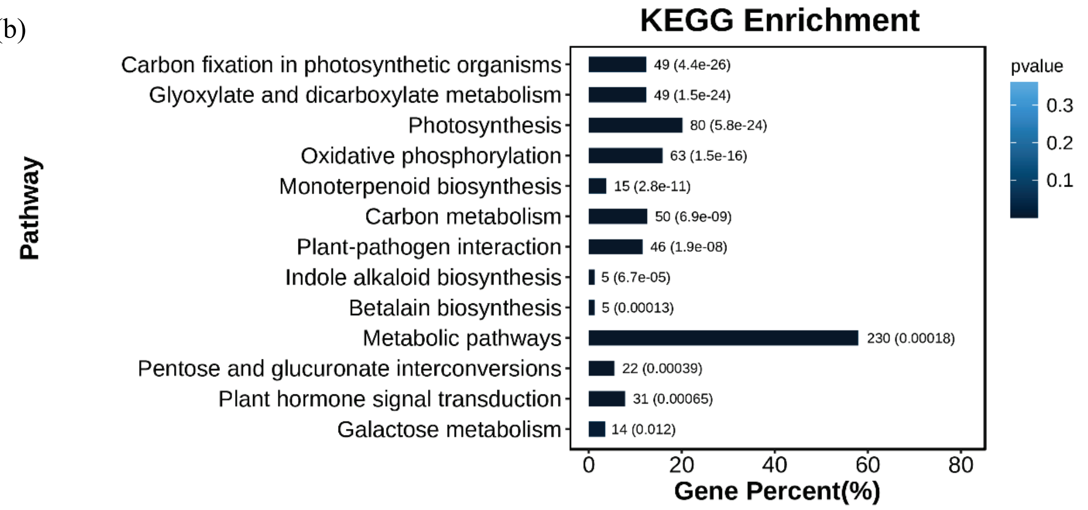

Figure S8. Visualization of results from (a) GO and (b) KEGG enrichment analysis of 2254 significantly expanded genes in *A. pentaphyllum*. BP = biological process, CC = cellular component, MF = molecular function.

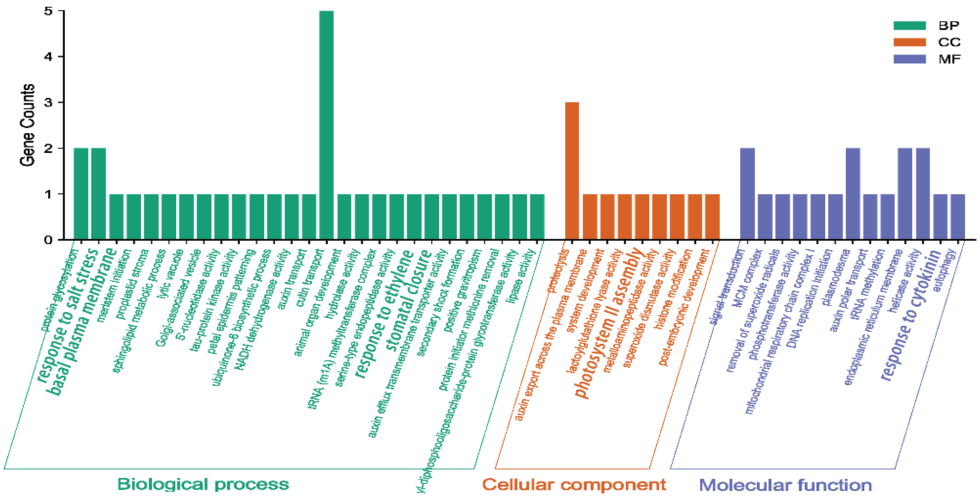

Figure S9. GO enrichment analysis of positive selected genes in *A. pentaphyllum*.

(a)

(b)

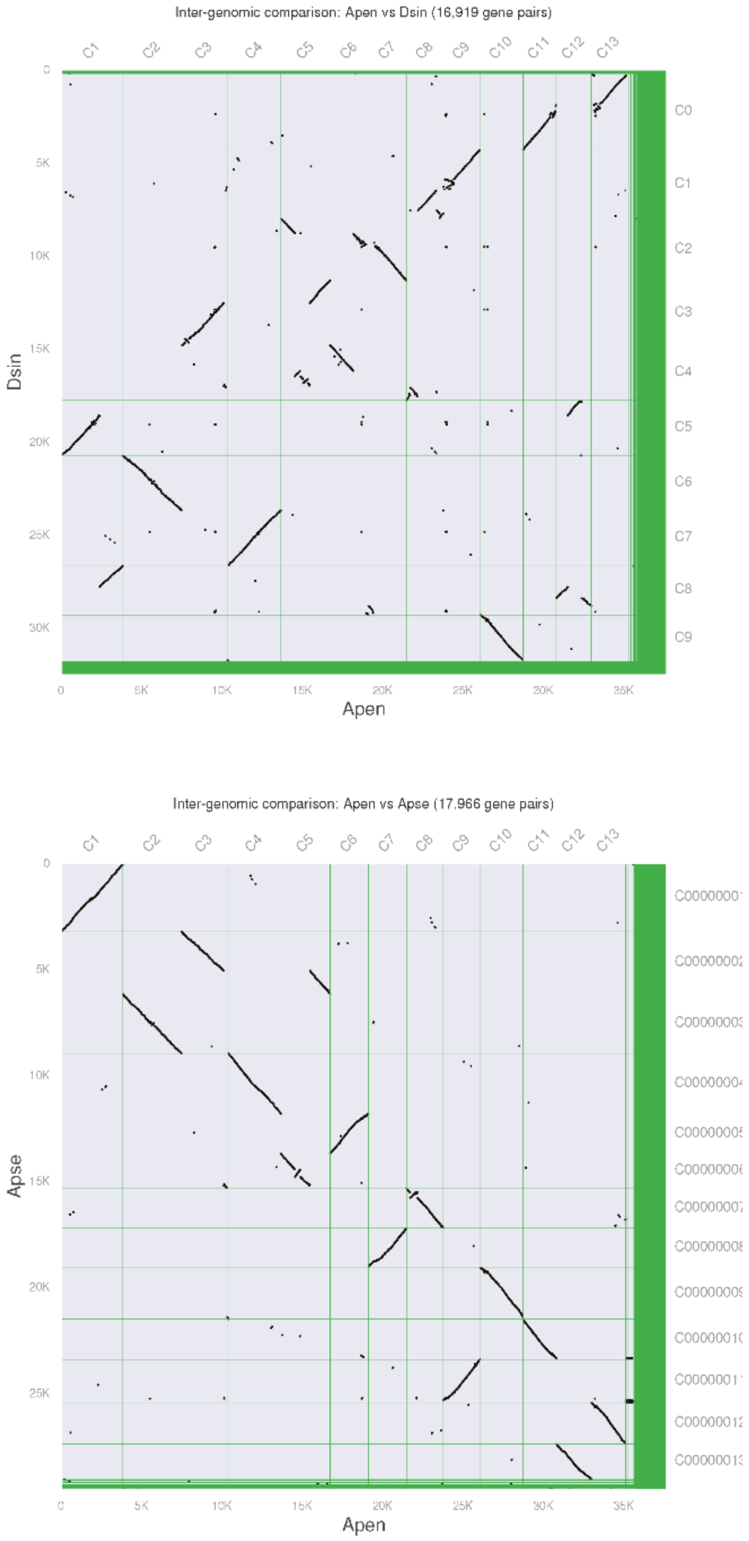

Figure S10. Dot plots of synteny blocks among *A. pentaphyllum*, (a) *A. yangbiense*, and *D. sinensis*.

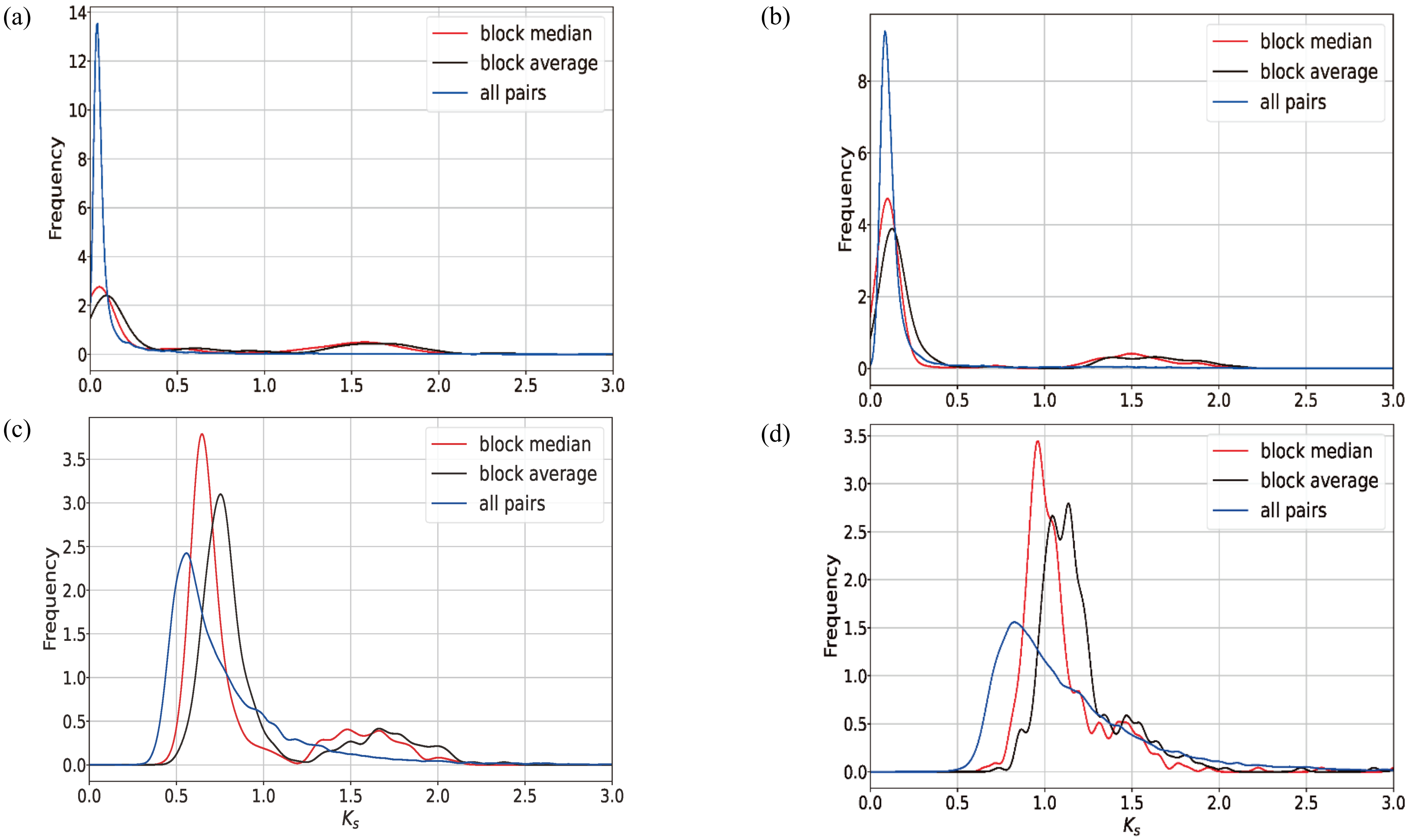

Figure S11. Synonymous substitution rate (*Ks*) distribution maps of synteny blocks between and within species. (a) *A. pentaphyllum vs A. yangbiense*, (b) *A. pentaphyllum vs D. dyeriana*, (c) *A. pentaphyllum vs C. sinensis*, (d) *A. pentaphyllum vs V. vinifera.*

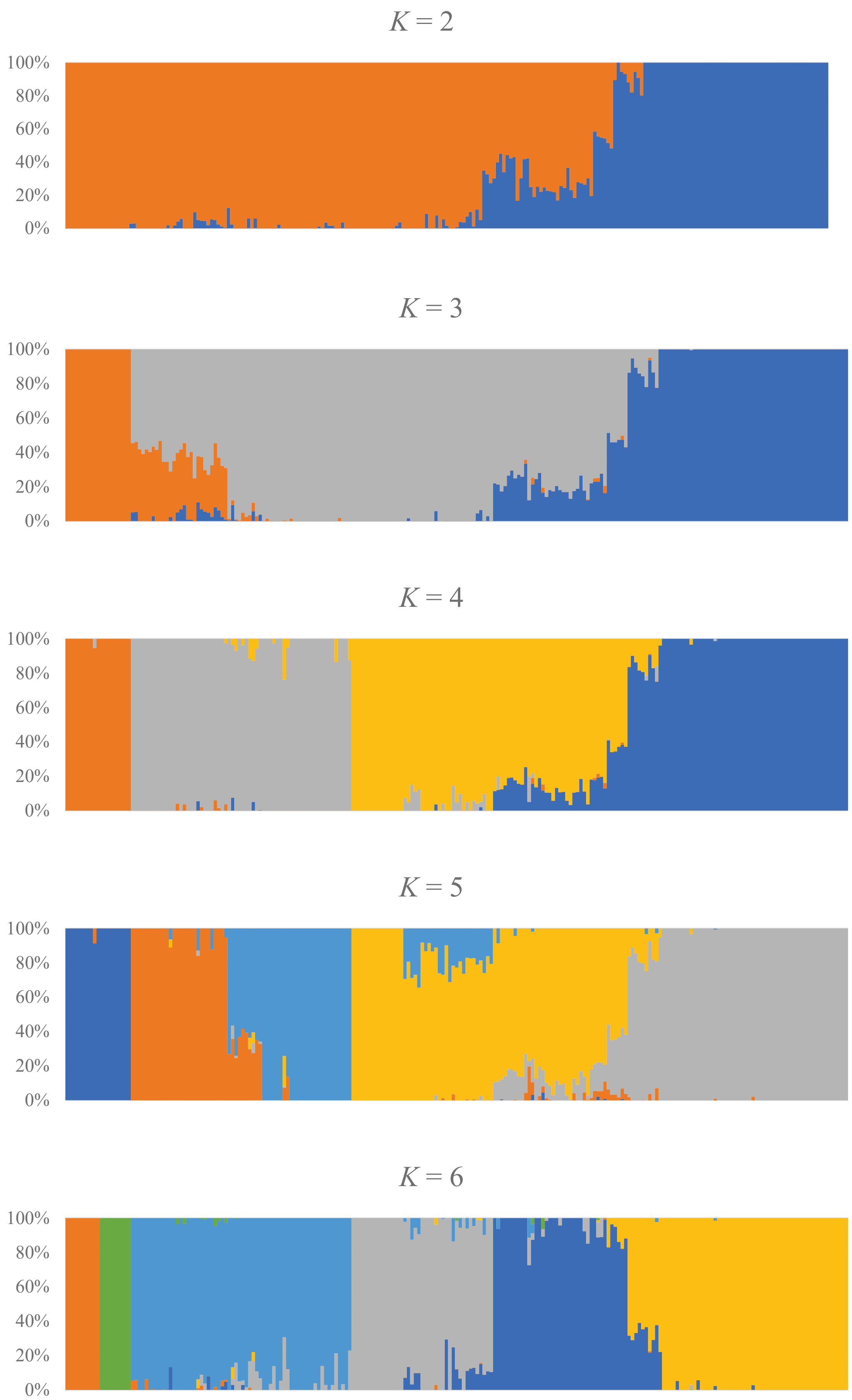

K = 2
K = 3
K = 4
K = 5
K = 6
100%
80%
60%
40%
20%
0%

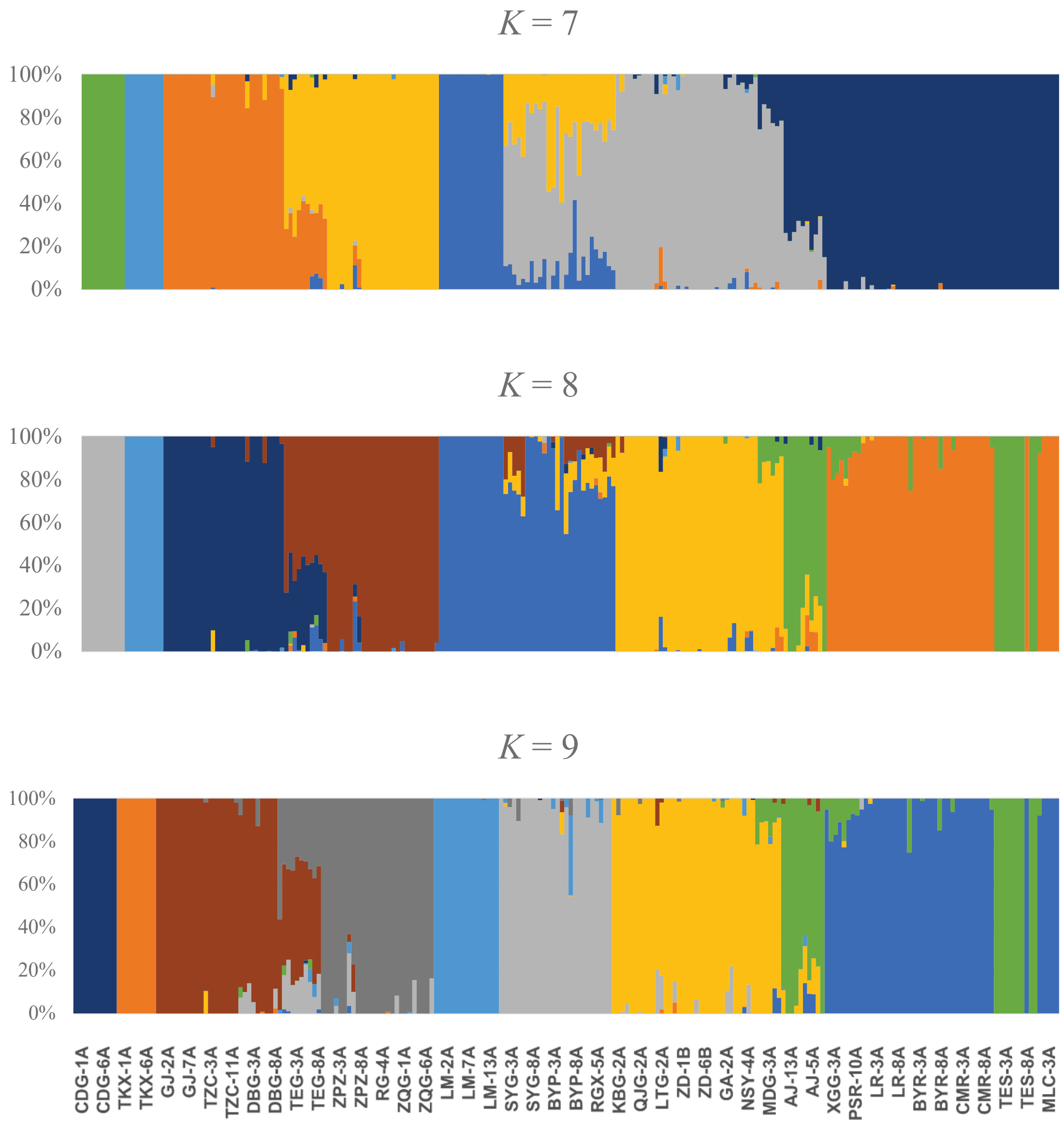

Figure S12. Population structure of 227 *A. pentaphyllum* individuals at $K = 2$ to $K = 9$.

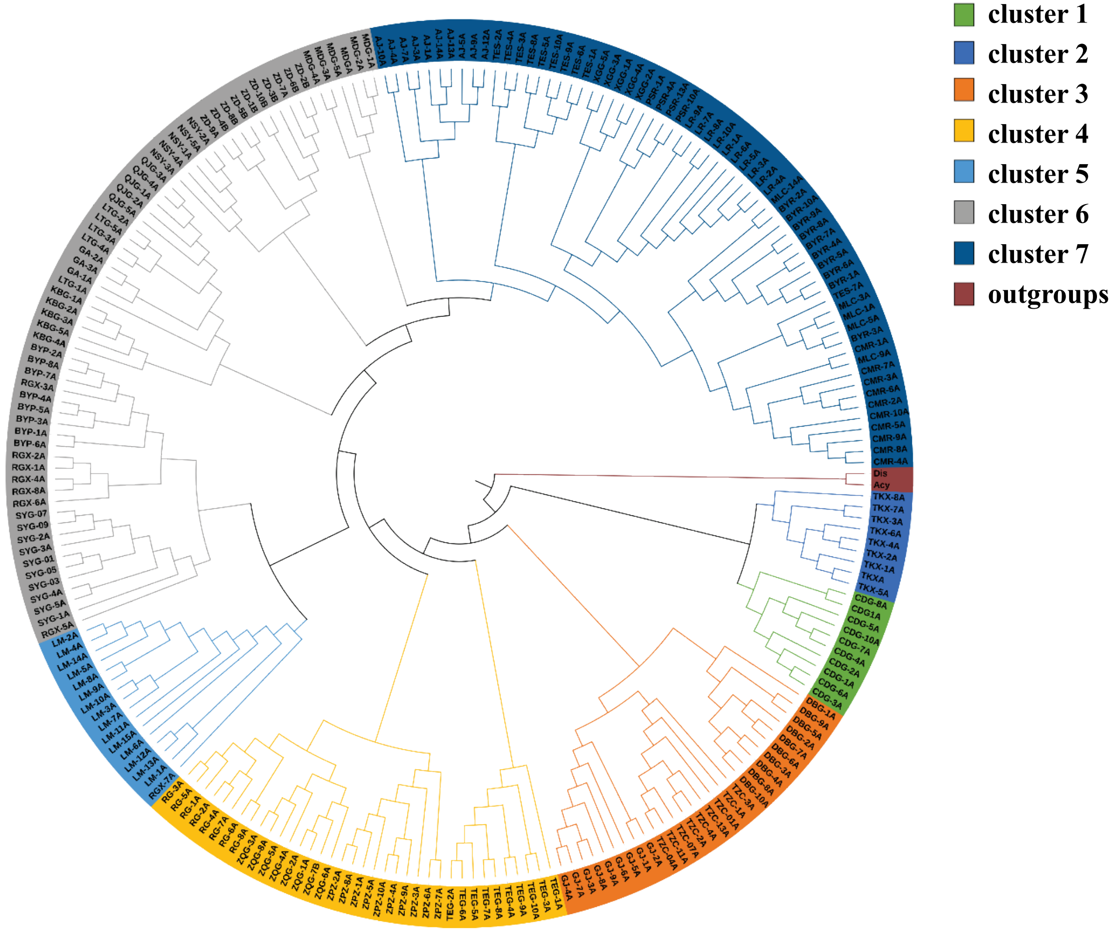

Figure S13. The phylogenetic tree of *A. pentaphyllum* was constructed based on Dataset 4 (different colors represent different clusters at $K = 7$).

(a)

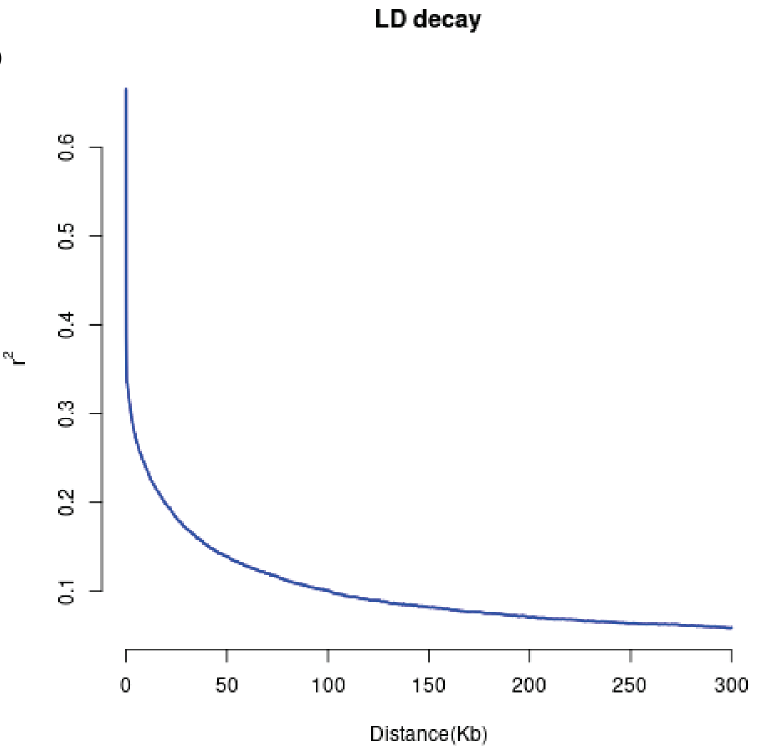

(b)

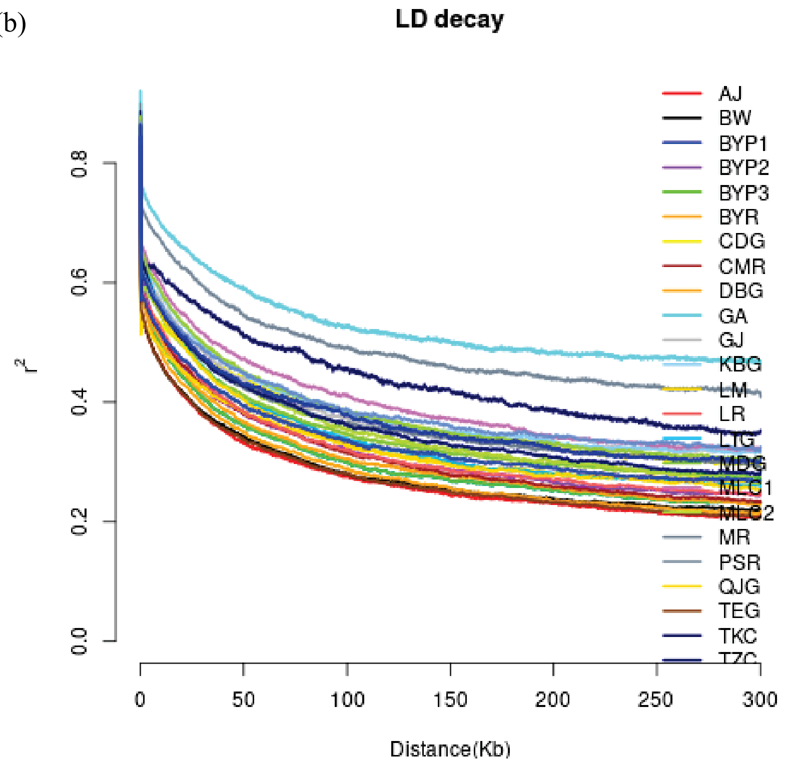

Figure S14. Genome-wide linkage disequilibrium (LD) decay in *A. pentaphyllum* among 28 separate populations (a) and considering the ten populations as a whole (b).

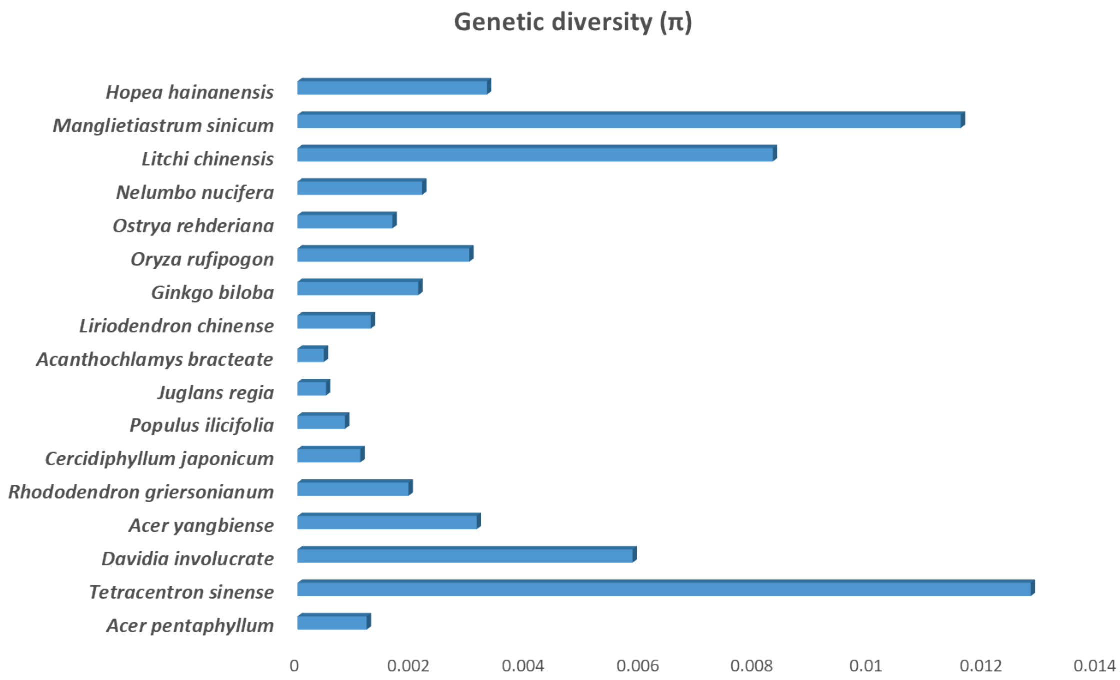

Figure S15. Genome-wide nucleotide diversity (π) for 17 threatened woody species.

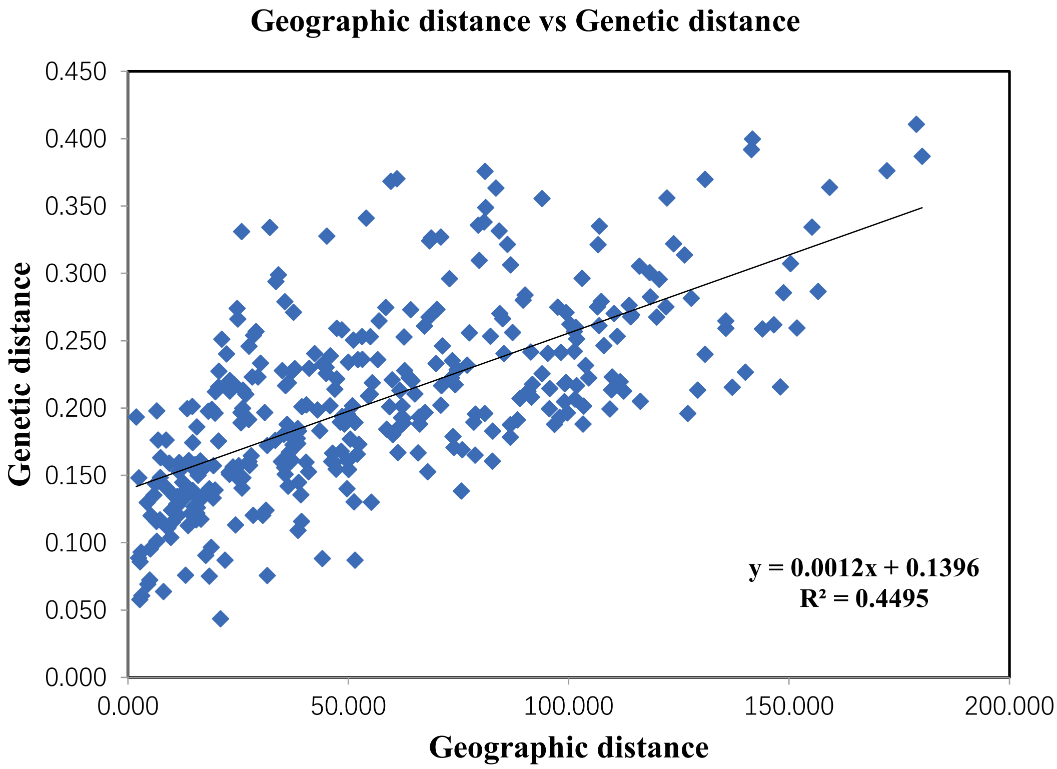

Figure S16. Mantel test plot of genetic and geographic distances for *A. pentaphyllum.*

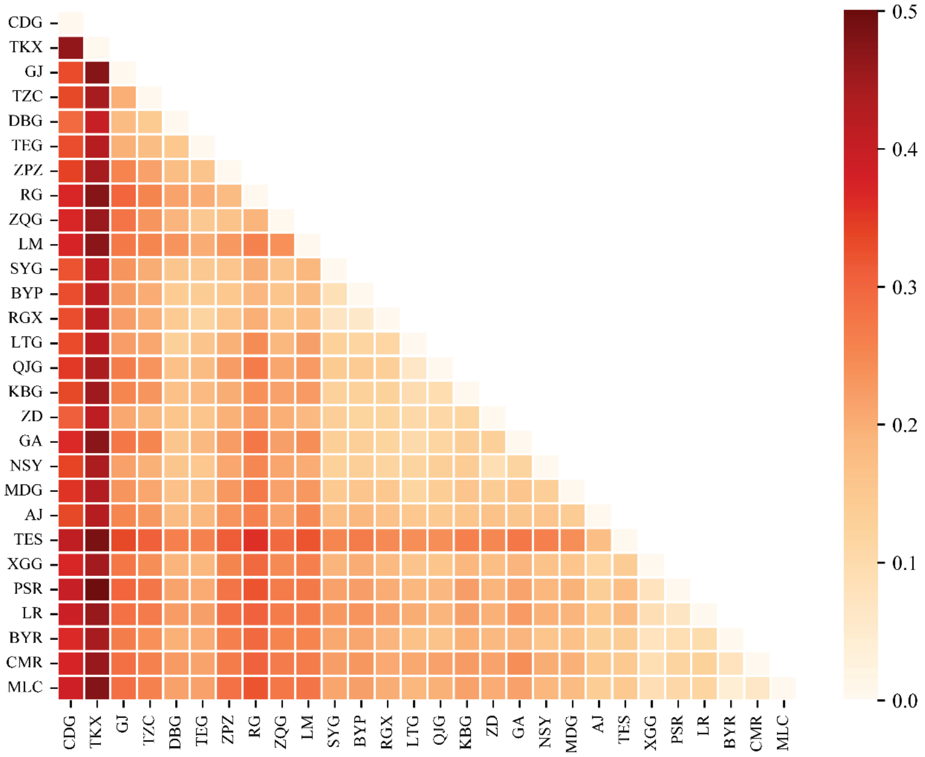

Figure S17. Genetic differentiation level (F_{ST}) of each population for *A. pentaphyllum.*

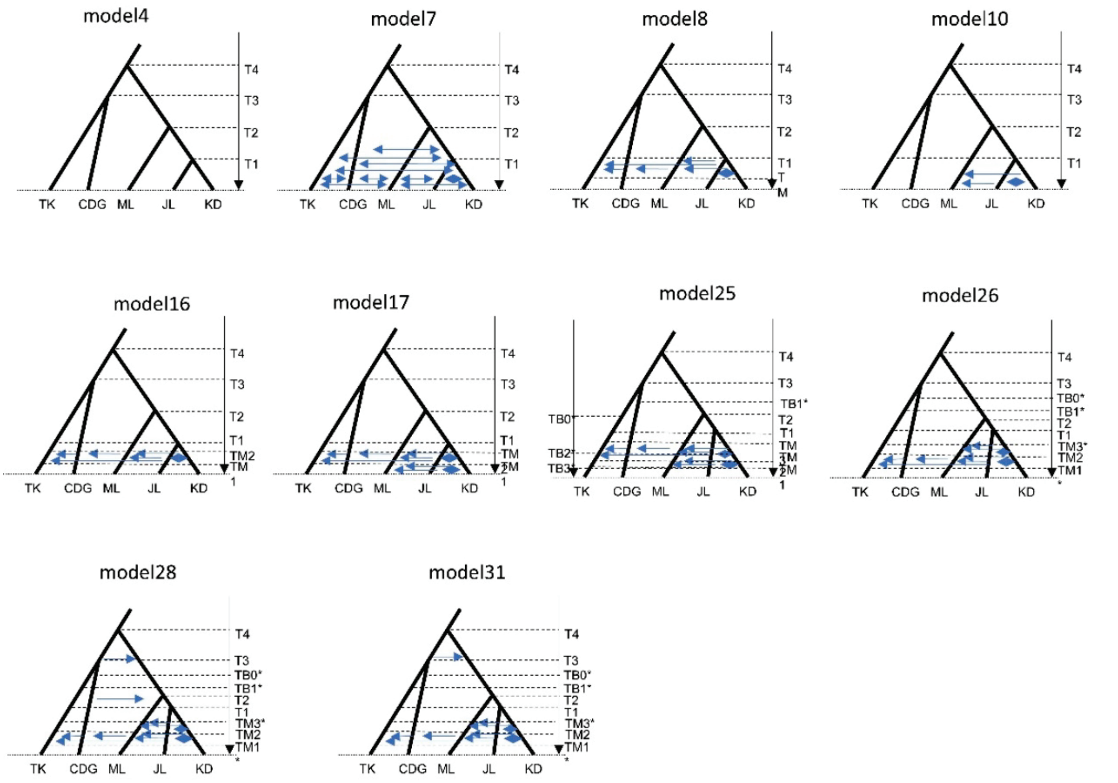

Figure S18. Representative scenarios tested in this study. Asterisks besides parameters represent changes in population sizes.

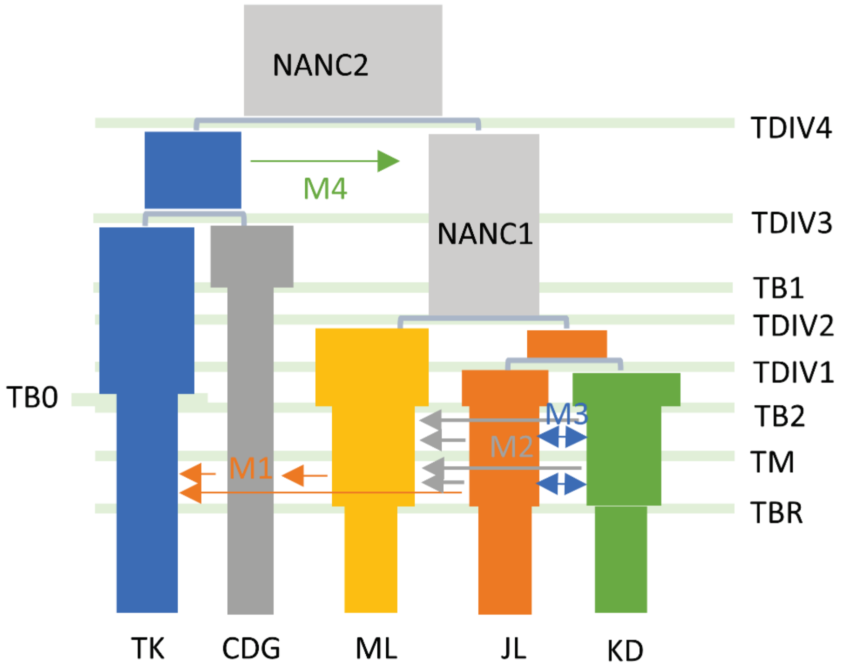

Figure S19. Illustration of the best-fit model with parameters names.

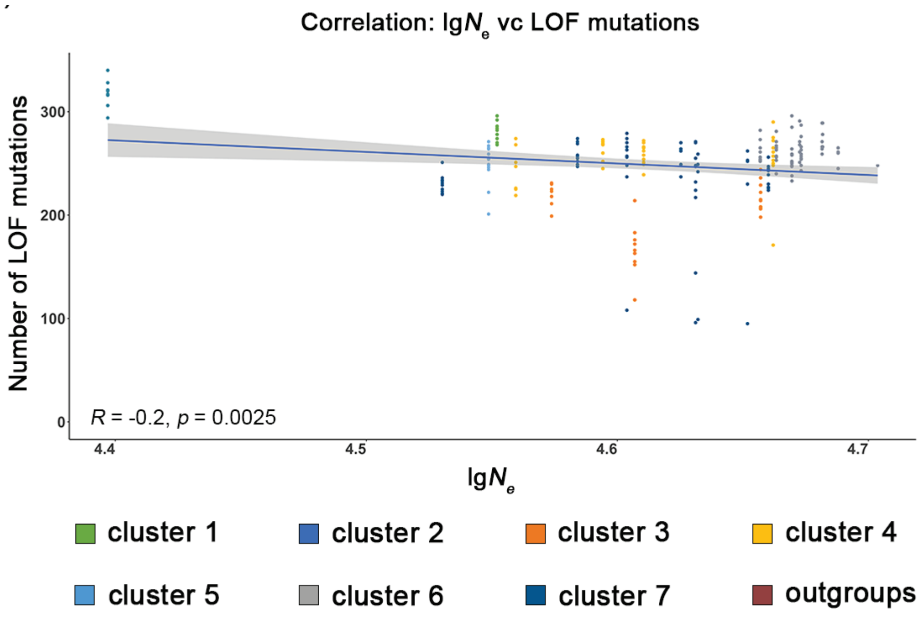

Figure S20. Correlation analysis between the number of extreme deleterious mutations (LOF) and the effective population size (N_e) across the 28 populations of *A. pentaphyllum.*

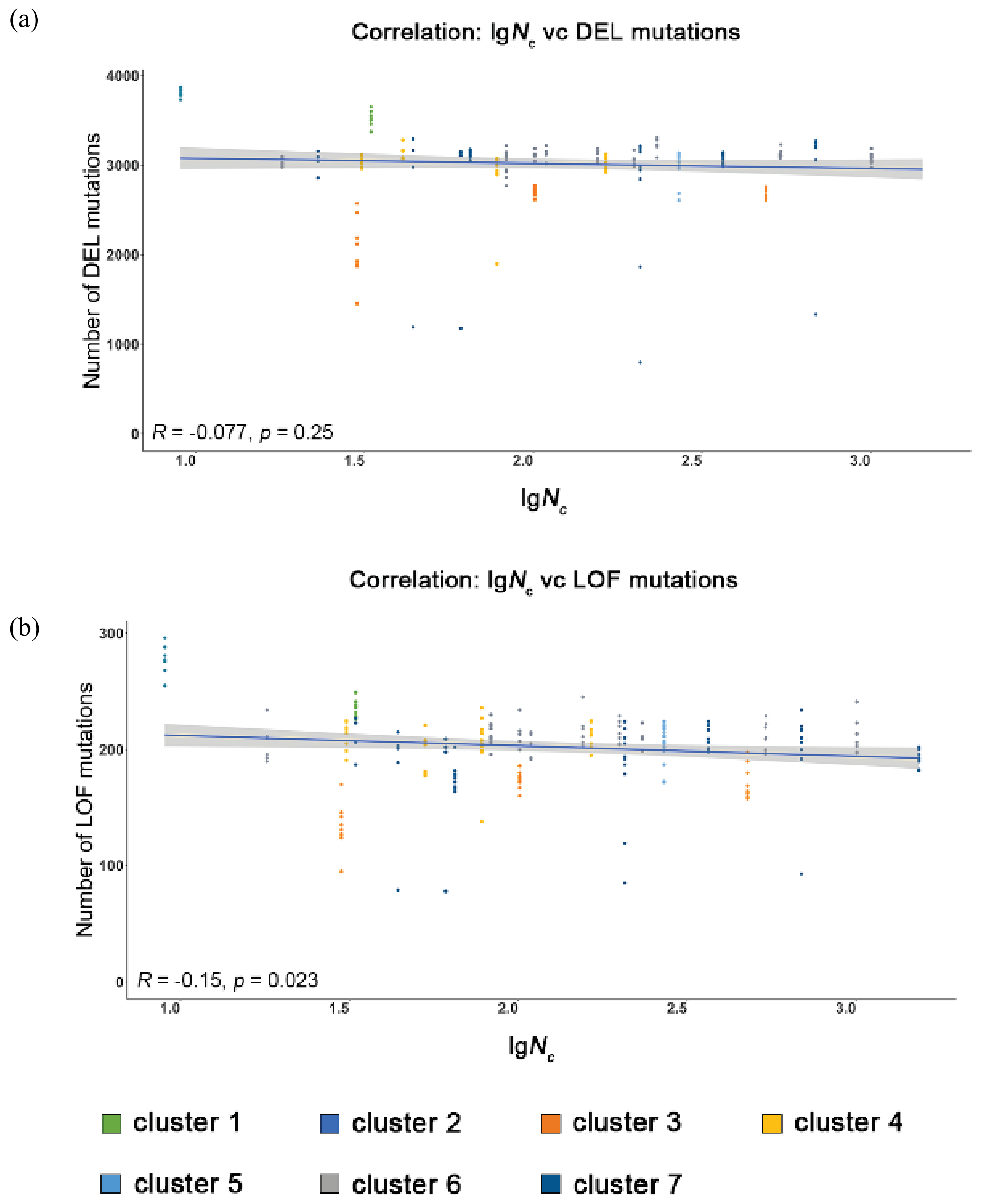

Figure S21. Correlation analysis between the census population size (N_c) and the number of deleterious mutations of (a) DEL and (b) LOF across the 28 populations of *A. pentaphyllum*.

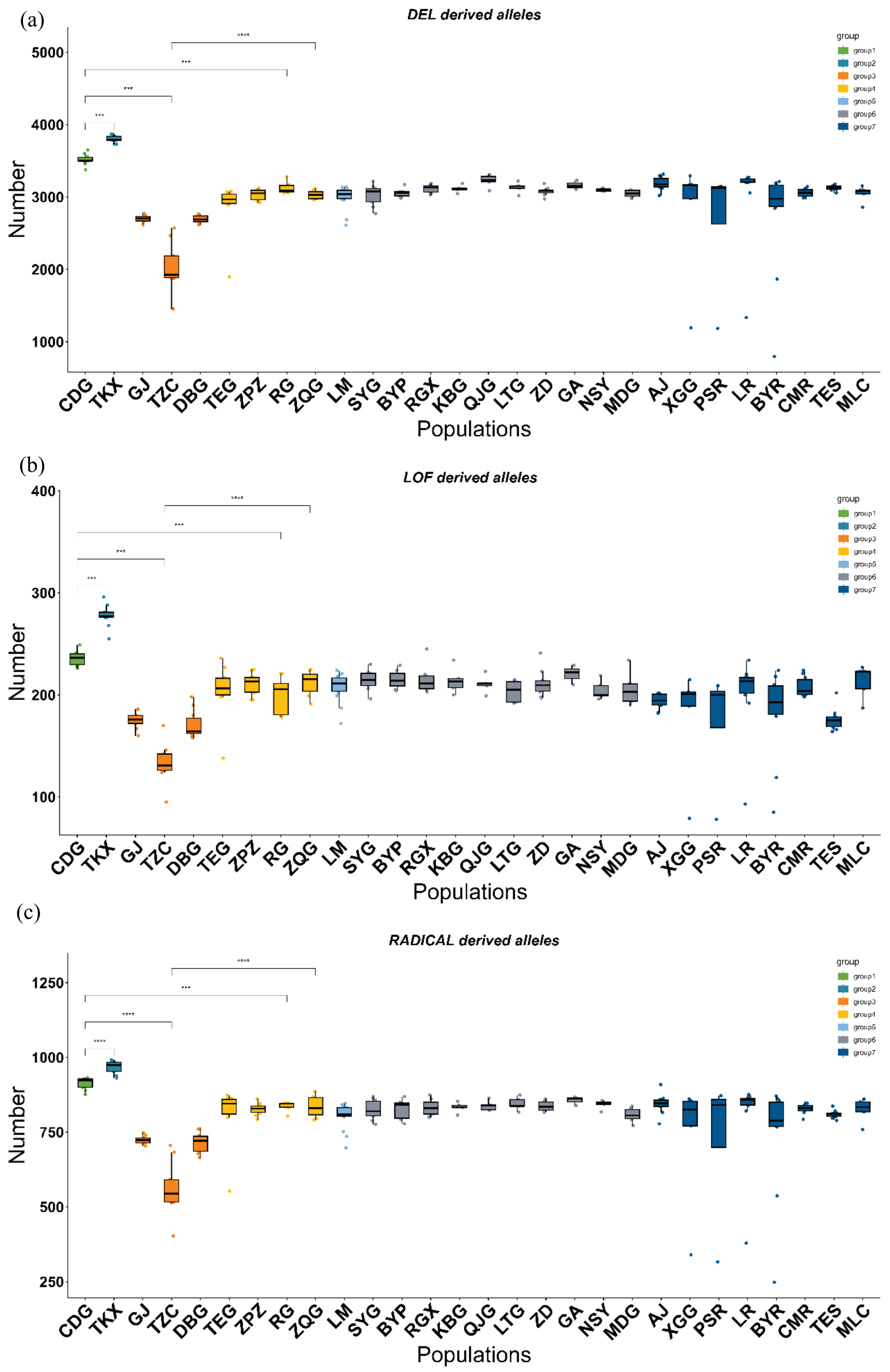

Figure S22. Number of (a) DEL, (b) LOF, and (c) RADICAL mutations among 28 populations of *A. pentaphyllum*.

(a)

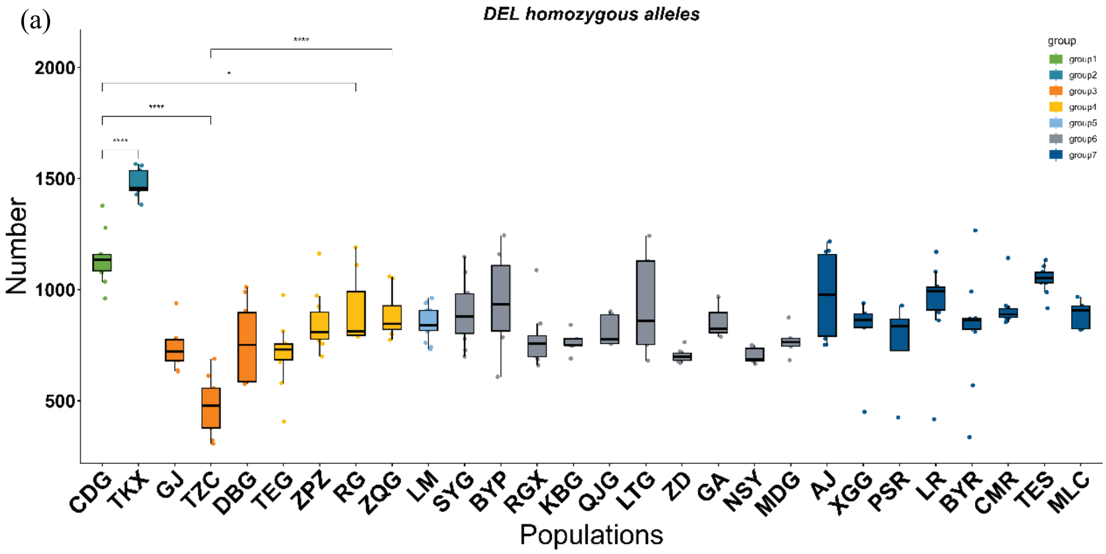

(b)

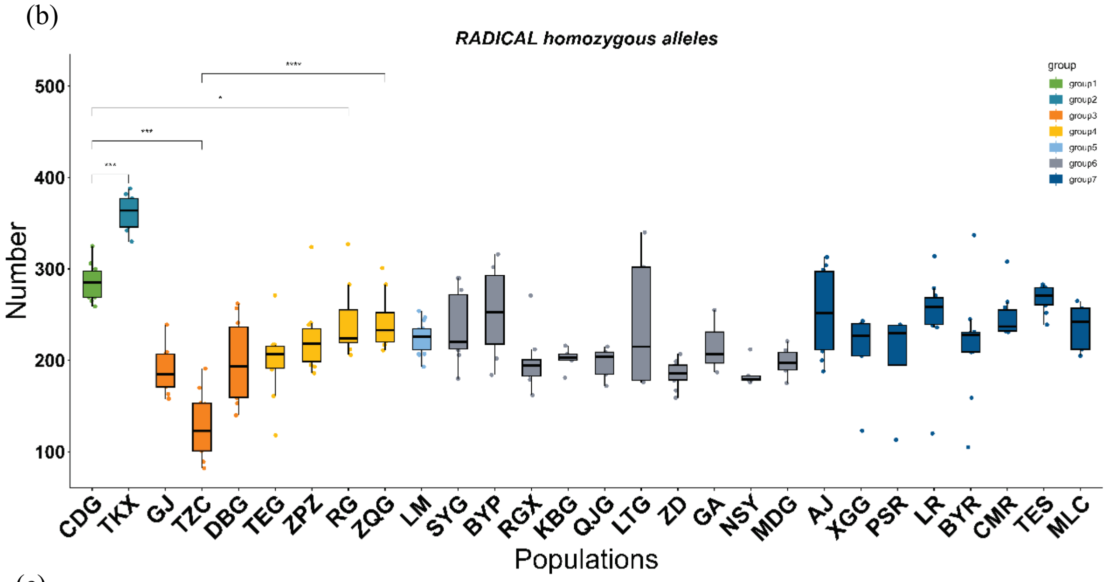

(c)

Figure S23. Number of homozygous (a) DEL, (b) LOF, and (c) RADICAL mutations among 28 populations of *A. pentaphyllum*.

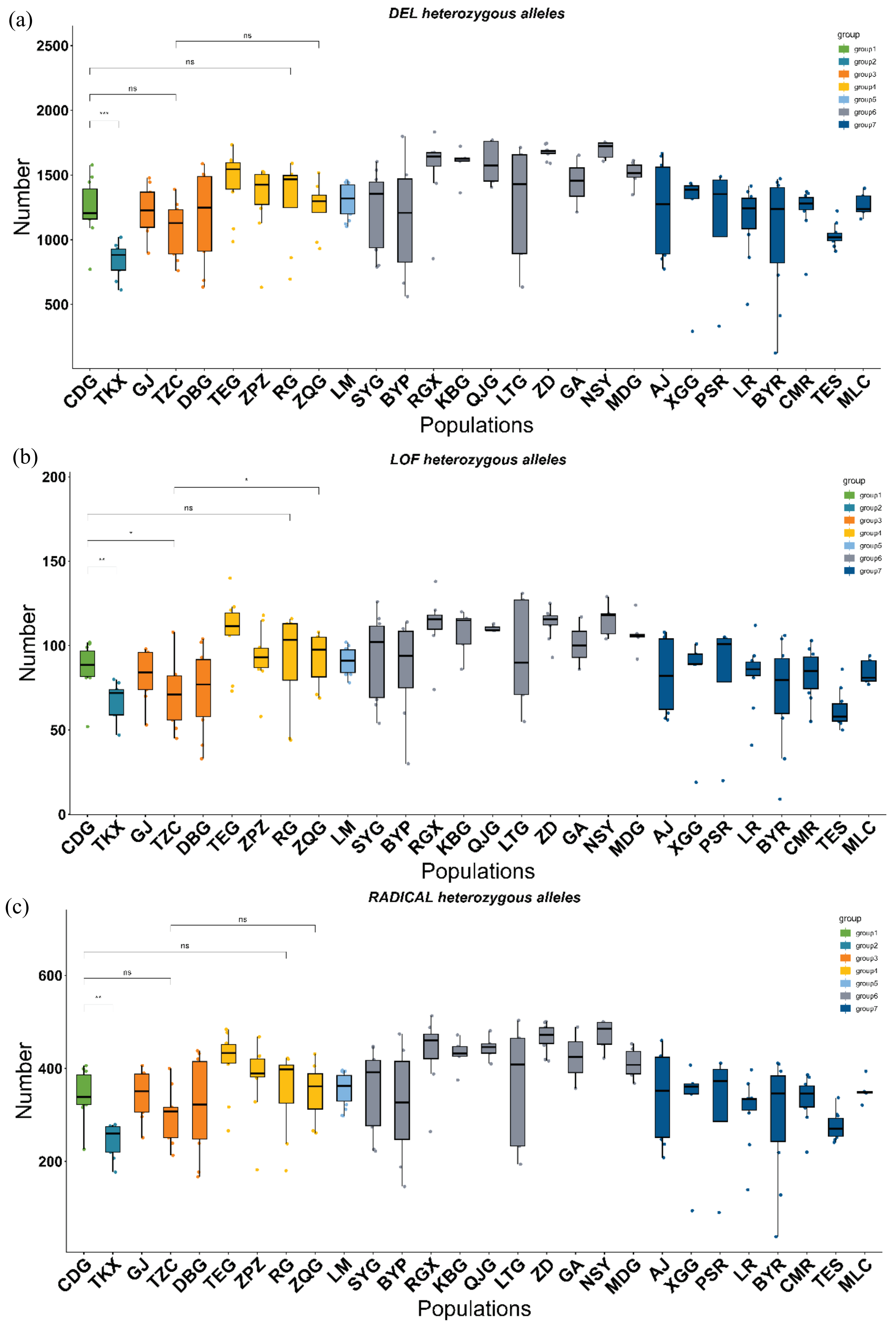

Figure S24. Number of heterozygous (a) DEL, (b) LOF, and (c) RADICAL mutations among 28 populations of *A. pentaphyllum.*

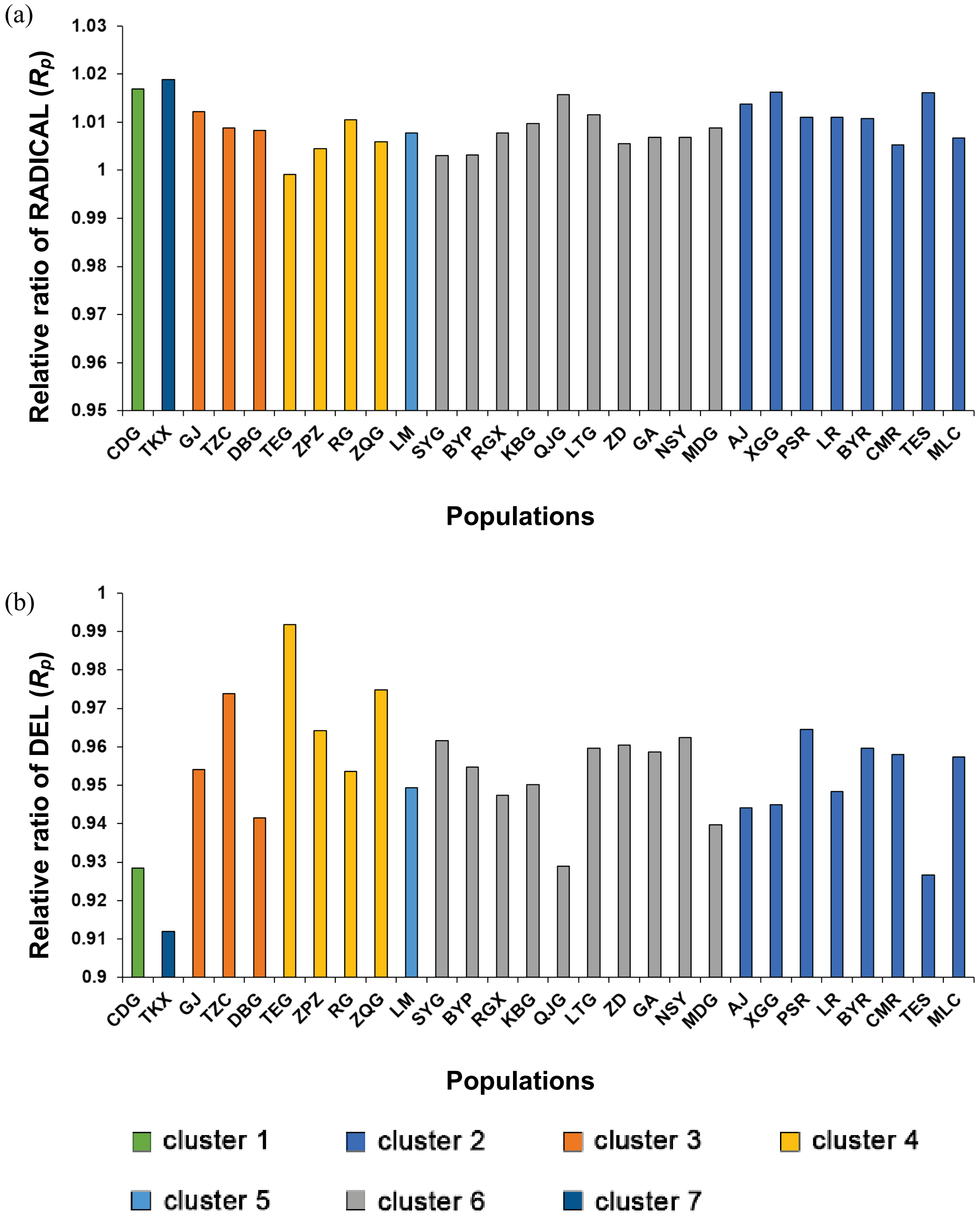

Figure S25. The relative ratio (R_p) of the mean derived alleles frequency (p) for (a) DEL and (b) RADICAL mutations.
